# Supplementary material for: What is the impact of targeted therapies given within phase I trials on the cognitive function of patients with advanced cancer: a mixed-methods exploratory study conducted in an early clinical trials unit
Source: BMJ Open. 2022 Nov 28;12(11):e050590. doi: 10.1136/bmjopen-2021-050590 (PMC9710342; doi:10.1136/bmjopen-2021-050590)
Supplement: Supplementary data [file bmjopen-2021-050590supp001.pdf]

Appendix A Interview Schedule

Interviewer to introduce themselves to the participant, clarify that they know why they are here and that consent still stands. Give examples of phrasing of questions and suggested idea of how long the interview will last. Remind the participant that they can stop the process if they wish. Recheck that they are happy for the audio recording to start, and they are ready to begin the interview.

Using open ended questions, the Interview will cover the following at least but not exclusively the following areas:

Memory

Concentration

Ability to speak/articulate/communicate

General Health

Impact of trial treatment/schedule

Mood

The first two questions will be general to help the patient to start talking and feel relaxed in the environment

1) Tell me how you have been since you started the trial

2) Can you tell me of any changes you have noticed since starting the trial?

If the participant mentions any cognitive or related symptoms, they will be asked to expand on this, using such phrases such as

- **“**Can you tell me a bit more about that?”
- “What was that like for you?”
- “How did you deal with that? “

If not questions regarding domains of cognitive function will be asked – examples of this include:

“Tell me about how your memory has been since you started the trial”

“tell me how your concentration has been since starting on the trial? “

"Have you noticed any differences in speaking or finding the right words sometimes?"

"Have you noticed any changes in your ability to think straight or work things out?"

"Have you had any difficulty relating to this? "

"How has your mood been since starting on the trial drug?"

If any changes or symptoms are discussed ask patient to elaborate – when did it start, how does it manifest/feel/ present? – Characterise the symptom with the participant onset, severity, duration, improvements, deterioration etc. Examples of questions to elaborate on symptoms are:

"How did you first notice this?"

"Tell me a bit about what happened to you "

"How did you feel when this was happening?"

Other questions will then cover the areas that are relevant to the investigation of this study. If patients have discussed specific symptoms the following questions will be asked in relation to this.

"Tell me how this has impacted on your life?"

"Has this affected you at work/home/ your relationships with others in any way?"

Once the impact and effect on certain areas has been established try to prompt further to explore how this made the patient feel and how they cope with this.

"Did you try anything to help the situation? "

"Was this a difficult thing to cope with?"

Dependant on what the interviewee discusses during the interview, complete with an exploration of strategies or useful interventions that have or may have helped covering:

"Would they have been anything useful to know about pre- treatment to help you prepare for any of these symptoms?"

"Tell me about any strategies that you have used yourself to help you overcome these problems?"

"tell me of anything the team here could have done to help you manage the ……. You discussed before "

The last two questions are regarding the acceptability of the assessments performed for this study- these questions will be asked to all participants.

"How have you found the extra cognitive assessments that have been performed for this study?"

"Would you be happy to have these assessments performed alongside treatment as the norm?"

End the interview by checking if there is anything that the participant wants to add. Ask if they are happy for the interview to be used. Thank the participant for taking part.
