# Supplementary material for: What is the impact of targeted therapies given within phase I trials on the cognitive function of patients with advanced cancer: a mixed-methods exploratory study conducted in an early clinical trials unit
Source: BMJ Open. 2022 Nov 28;12(11):e050590. doi: 10.1136/bmjopen-2021-050590 (PMC9710342; doi:10.1136/bmjopen-2021-050590)
Supplement: Author's manuscript [file bmjopen-2021-050590.draft_revisions.pdf]

BMJ Open

BMJ Open is committed to open peer review. As part of this commitment we make the peer review history of every article we publish publicly available.

When an article is published we post the peer reviewers' comments and the authors' responses online. We also post the versions of the paper that were used during peer review. These are the versions that the peer review comments apply to.

The versions of the paper that follow are the versions that were submitted during the peer review process. They are not the versions of record or the final published versions. They should not be cited or distributed as the published version of this manuscript.

BMJ Open is an open access journal and the full, final, typeset and author-corrected version of record of the manuscript is available on our site with no access controls, subscription charges or pay-per-view fees (http://bmjopen.bmj.com).

If you have any questions on BMJ Open's open peer review process please email info.bmjopen@bmj.com

The Impact of targeted therapies given within Phase I trials on the cognitive function of patients with advanced cancer.

Journal:	*BMJ Open*
Manuscript ID	bmjopen-2021-050590
Article Type:	Protocol
Date Submitted by the Author:	23-Feb-2021
Complete List of Authors:	Stapleton, Sarah ; Royal Marsden Hospital Sutton, Drug Development Unit ; University of Southampton, Faculty of Health Sciences<br>Darlington, Anne-Sophie; University of Southamptom, Faculty of Health Sciences<br>de Bono, J. S.; Royal Marsden NHS Fdn Trust; Institute of Cancer Research Division of Cancer Therapeutics<br>Wiseman, Theresa; The Royal Marsden NHS Foundation Trust, ; University of Southampton, Faculty of Health Sciences
Keywords:	CLINICAL PHARMACOLOGY, Adult oncology < ONCOLOGY, Delirium & cognitive disorders < PSYCHIATRY, Clinical trials < THERAPEUTICS

SCHOLARONE™
Manuscripts

Cognitive Function Protocol for submission to BMJ

Title: The Impact of targeted therapies given within Phase I trials on the cognitive function of patients with advanced cancer.

Short Title: Cognitive Function and Phase I cancer drug trials

Authors:
Sarah. E. Stapleton (1) Prof. Anne-Sophie Darlington (2) Prof. Johann De-Bono (1,3) Prof.Theresa Wiseman (1)

1 Royal Marsden Hospital Downs Rd Sutton Surrey Sm2 5PT

2 University of Southampton University Rd Southampton SO17 1BJ

3 Institute of Cancer Research 15 Cotswold Rd Sutton SM2 5NG

Corresponding Author: Sarah Stapleton Royal Marsden Hospital Downs Rd Sutton Surrey SM2 5PT sarah.stapleton@rmh.nhs.uk

Keywords: Drug Development , Early Phase I Clinical Trials, Cognitive function, Cognitive impairment, Cognitive assessment

For peer review only

Cognitive Function Protocol for submission to BMJ

Abstract

Background: Novel therapies such as small protein molecule inhibitors and immunotherapies are tested in early phase trials before moving to later phase trials and ultimately standard practice. A key aim of these types of early clinical trial is to define a toxicity profile, however the emphasis is often on safety with measurements of organ toxicity. Other subjective side effects are often not reported or measured formally within the trial protocols. The concern from clinical practice is that cognitive toxicity which is not regarded in this context as a metric of toxicity is poorly studied and may be under-reported. This could lead to toxicity profiles treatments not being fully described and patients with unmet need in terms of acknowledgement and support of symptoms. This protocol outlines a framework of an exploratory study with feasibility aspects to investigate the impact and experience of cognitive changes for patients on Phase I trials.

Sample : 30 Patients with advanced cancer who are participating in Phase I trials of novel targeted therapies in the Early Clinical Trials Unit of a specialist Cancer Centre.

Methods: This is a mixed methods study combining quantitative and qualitative approaches. A test battery of validated cognitive assessments will be taken alongside patient reported outcome measures at three time-points from baseline, day eight and day 28 post start of treatment. At day 28 a semi structured interview will be conducted and the narrative thematically analysed. Results will be integrated to offer a comprehensive description of cognitive function for in this patient group.

Discussion: This will be the first study to introduce formal cognitive assessments in a Cancer Phase I trial context, it is has to potential to highlight previously unreported side effects and more importantly unmet need in terms of care for our patients who are participating in the trials.

Strengths and Limitations

- This is the first study to investigate cognitive function longitudinally during the course of a Phase I trials.
- The findings of the trial will establish feasibility of conducting more formal and consistent cognitive testing in early clinical drug trial protocols.
- Describing the symptom for patients in this group will help to identify unmet need in terms of support and management.
- Recommendations of the International Cancer and Cognition Task Force to harmonise research in this field were adopted in the trial design.
- Due to the exploratory nature and feasibility aspects of the study, the sample size is not statistically powered. A future study would benefit form a powered calculated sample size to demonstrate any treatment related change with more certainty.

For peer review only - http://bmjopen.bmj.com/site/about/guidelines.xhtml

Cognitive Function Protocol for submission to BMJ

In Cancer drug development advances in the understanding of genetics and the biochemical pathways involved in cancer have helped researchers to rationally design “molecularly targeted drugs”. These novel targeted therapies and immunotherapies are first tested clinically in patients within Phase I trials in cancer centres. Due to the experimental nature of Phase I trials and the fact that little is known about the toxicities, patients need to have exhausted all standard treatment options to participate. To contextualise, these patients will generally have advanced cancer pre-treated disease and often a limited prognosis. It is a heterogenous patient population in terms of tumour types. However what the patients to have in common is a shared experience of the continuous cycle of treatment and disease progression to the point where there is no further treatment available. They will often have significant tumour burden with metastatic disease and a life limiting prognosis. These patients remain motivated to participate in Phase I trials of experimental therapies and despite having advanced progressive disease the criteria for participating in Phase I means the patients do have to have a certain level of wellness to meet eligibility requirements of specific Phase I protocols.

At this stage of drug development the main aims are to find the optimal dose, investigate the pharmacology and to define the toxicity profiles (1). When considering toxicity profiles of these potential new treatments the emphasis is on safety and this is reflected in many of the standard assessments in the trial protocols such as cardiology monitoring and renal and liver function tests (2). Clinicians and nurses involved in the conduct of Phase I trials do perform medical history taking and nursing assessments to gain a full perspective of other side effects reported but they are interpreted and recorded in a subjective manner usually using the EORTC Common Terminology Criteria as a grading tool (3). This means that side effects less easy to measure such as fatigue, pain and cognitive function may be assessed inconsistently or not at all if patients choose not to report.

This exploratory study investigates the impact on the cognitive function of these patients with advanced cancer, who are participating in Phase I trials. It is suspected that some of the novel targeted therapies tested within Phase I trials may have a negative impact on the patient’s cognitive function. Evidence from clinical practice suggests that patients are experiencing symptoms relating to cognitive function but this does not translate to reports of cognitive toxicity within clinical trial data. As cognitive function is not formally assessed the extent, nature or domain of cognitive function is not well described, therefore the impact on cognitive function for patients on Phase I trials is essentially unknown and there is a suggestion in literature that the difficulty in ascertaining

For peer review only

the relationship between cognitive function impairment and targeted therapies in cancer patients may be due to the underreporting of cognitive symptoms in clinical trials (4). Other studies support the idea that this symptom is under recognised and underreported by patients on treatment and participating in trials (5).

Rationale

Impairment of cognitive function in the field of traditional cytotoxic chemotherapy has been investigated and it is known that such a symptom impairs quality of life (6). Therefore if cognitive function was investigated and described in the context of patients on Phase I trials, it could provide a basis of knowledge on which to develop interventions and coping strategies with the aim of preventing or ameliorating the effects of this possible decline in cognitive function.

Cognitive function is described as the internal processes involved in making sense of the world and the capacity to make decisions (7). Such processes include attention, perception, learning, memory, language, problem solving, reasoning and thinking (8). These functions are defined in terms of domains that can broadly be divided into sensory and motor skills, memory, attention and concentration, executive function (which includes reasoning and problem solving) and language and verbal skills (9). Whilst the domains overlap in terms of function each can be assessed using validated cognitive assessment tools. Impaired cognitive function in cancer patients can present as loss of memory, difficulty with word finding, loss of concentration and difficulty in task switching (10). Even though cognitive function is not assessed as part of the Phase I trials, anecdotal reports from some patients suggest that they experience some deterioration whilst on the trial often temporal to dosing of drug.

For patients this has implications in that they may have a symptom they feel unable to discuss. This in turn can mean that they do not receive any help or support regarding the problem and can be isolating and frightening for the patient (11). Not having the symptom acknowledged or having no information to warn about the possibility of it happening has been reported to be one of the most distressing and difficult things for patients to cope with (11,12).

Implications on a broader scale will mean that better defined toxicity profiles will improve understanding of consequences of treatments. This will assist in developing interventions that help

For peer review only

patients make informed choices about having certain treatments and be better equipped to cope with the consequences of them. This may be as simple as recognition of the potential problem with information and preparation for the patient or the implementation of strategies to help with symptoms such as compensatory memory techniques, rehabilitation and relaxation (13,14,15).

In terms of relevance the numbers of patients initially receiving such treatments in Phase I trials may be relatively small, usually tens to hundreds. Nevertheless for these patients with a limited prognosis issues regarding side effects are important. Once a drug has gone through the phases of testing and is licensed for practice the numbers of patients receiving these treatments could then become thousands (16). The number of cancer patients participating in clinical trials is increasing (17). Therefore it is important to find out accurately what is happening to patients on clinical trials so they can be supported with the management of side effects such as diminished cognitive function. In order to begin to address this and improve the care and understanding of patients on Phase I trials this proposed study will be the first exploration using formal assessment of cognitive function in this context.

Evidence Gap

Early work in chemotherapy related cognitive impairment highlighted a disparity in trial designs and some limitations in existing research in this field such as the use of cross sectional trial design offering no baseline from which to measure change. Lack of consistency in the type of cognitive assessment methods used in the trials also lead to broad differences in reporting of the prevalence of cognitive impairment. This led to a group of expert cognitive researchers founding an international multidisciplinary group known as the International Cancer and Cognition Task Force (ICCTF). The ICCTF was founded in 2004 and one of the aims was to produce guidelines to harmonise study design. Their recommendations were to include pre-treatment assessment, longitudinal follow up, the use of a control group and a core set of cognitive assessment tests when investigating cognitive function in cancer patients (18). All recommendations will be adopted for this protocol except the use of a control group. It would be difficult to match a control for this patient group as the main reason that patients do not enter a Phase I trial is due to a deterioration in clinical condition. Therefore patients in a comparable situation would likely be receiving palliative care only and it may not be appropriate to ask them to participate in order to provide a control.

The subsequent studies that followed these guidelines continued to show associations between systemic chemotherapy treatment and a decline in cognitive function as well as discovering that

For peer review only

patients with cancer often have a degree of cognitive impairment at baseline when compared to matched healthy controls. (19,20,21,22,23,24,25,26). The studies also demonstrated the manifestation of cognitive deficits which presented as loss of concentration, memory, verbal fluency and executive functioning which includes higher order cognitive abilities of planning and abstract thinking. Due to the longitudinal design some have been able to demonstrate compelling evidence of dose dependent relationships between treatment and severity of diminished cognitive function (20,24).

Some of the studies also highlighted the value of collecting the patient's own perception of cognitive function as they showed that this can vary from the validated cognitive assessment measures, (27,28). What is not addressed in many of these studies is the meaning to the patient in terms of how it may affect their lives. Just a small number of qualitative studies have been conducted exploring the impact of cognitive function on the quality of life of patients on traditional cytotoxic chemotherapy. They show that there is a negative effect on work, sometimes ability to return to previous occupations and effects on emotional, social and psychological aspects of daily life (29)

A very limited number of published Phase I clinical trials report cognitive toxicity and this seems to be where cognitive symptoms are severe enough to be defined as a dose limiting toxicity. A dose limiting toxicity is a pre-determined side effect criteria, usually severe that is outlined in the protocol and is directly attributable to drug. If a patient experiences a dose limiting toxicity it influences the direction of at trial. An example of this is a Phase I trial of a PI3K and Mtor inhibitor in combination that reported that 7.1% (n=28) patients experienced memory loss, confusional state, attention deficit and agitation that was attributable to the investigational medicinal product (IMP's). A further 21% of patients on this study experienced depression, with one patient experiencing a psychotic episode with suicidal ideation. This was classified as a dose limiting toxicity and deemed to be attributable to drug (30). Because no formal cognitive assessments were incorporated into the study design, the severity and duration of the memory and attention loss is unclear. A small number of other Phase I studies report cognitive dysfunction as part of the toxicity profile of drugs tested in Phase I. The symptoms are not well described in any of the publications and are reported using umbrella terms of cognitive events which include descriptions of mood alteration and concentration deficits in trial publications (31,32,33).To date there are no Phase I trials utilizing formal cognitive assessments within the trial protocols. This demonstrates the lack of adequate cognitive assessment at the early Phases of drug development.

For peer review only

In summary, there is a lack of evidence to show the effects on cognitive function for patients on early Phase I clinical trials. This has a two-fold consequence. Firstly that the side effects of the drugs may not be as well characterized and may only become apparent in latter stages of drug development or when a drug becomes standard practice. Secondly patients who are participating in Phase I trials and may have unmet need in terms of identification and support of symptoms of cognitive impairment. This research protocol outlines an exploratory framework for a study utilizing mixed methods to both identify the existence of cognitive decline in these patients but also describe the impact this has on their everyday lives.

Research Question and study aims

3. Aims

The study is framed as an exploratory study and the overarching aim is to identify, characterise and gain an understanding of the effects on the cognitive function of patients on novel targeted therapies given within the context of Phase I trial. The study has an added feasibility aspect to gain an understanding of how acceptable and appropriate nurse led cognitive assessments are in this patient group due to the existing potential burden of the Phase I trial commitments. Therefore the overarching research question for the study is:

"What is the impact of the targeted therapies given within the context of a Phase I trial on the cognitive function of patients with advanced cancer?"

Essentially the research question to explore this can be broken down into four sub questions. The research sub questions are presented in a table 1 below with the methods that will help gain insight into the question alongside how the analysis will be conducted.

Insert Table 1 Here

Methods and Analysis

This is a mixed methods study design using both qualitative and quantitative approaches with equal weighting to gain a comprehensive description of cognitive function for patients with advanced cancer on a Phase I trial of a novel therapy.

For peer review only

Sample

A non-probability sampling strategy technique of convenience sampling will be used. Most of the patients entering a Phase I trial will be eligible for this study therefore this sampling technique will allow for the quickest access to patient recruitment. The patients will be recruited from the Phase I Clinical Trials Unit within a specialist cancer hospital. The patients attend the unit on a weekly basis. The patient group is heterogeneous in terms of tumour type, age and gender. However the similarities within the sample population will be the stage of disease, the types of previous treatment and context. Although a convenience sample will be used there will be some stratification in terms of gender. Due to sample size any stratification of other possible confounding factors such as age, educational status will not be possible. Age and educational status (as a key component of cognitive reserve) have been shown to have an influence of altered cognitive function. The sample split between male and female will aim to be equal as it is known that there are cognitive differences relating to gender (34). As this is an exploratory study with feasibility aspects, the sample size will be 30 patients.

Study Design

The following figure represents an overall schema of the study. The methods will be described in greater detail in the following section.

Insert Figure 1 Here

The following figure represents the patient's pathway in relation to the pathway of the Phase I clinical trial.

Insert figure 2 here

Quantitative Methods

This study follows the ICCTF recommendations of a core battery of validated cognitive assessments of memory, verbal fluency and executive functioning at baseline , day eight post start of trial treatment and day 28 post start of trial treatment. As well as the cognitive assessments that will be conducted by the primary researcher there will be patient reported outcome measures of fatigue , depression and anxiety and self- perception of cognitive function. The following table lists the methods of quantitative data collection tools and how they are used.

For peer review only

Insert table 2 Here

Qualitative Methods

The qualitative method of data collection will be a semi-structured interview which will take place at 28 days post start of treatment. The interview will be digitally audio recorded and transcribed verbatim. The purpose of the interview is to drill down into any issues identified by the quantitative assessments, but also to further illicit information regarding possible impact on the patient's life that this problem may have caused. The interviews are semi-structured but can be flexible so that they can be guided by any issues highlighted by the quantitative data collection.

The interview will be conducted by the primary researcher, in a private room and last for up to one hour. The participant will be told that they can have a break or stop the interview at any time. Interviewing patients on potentially sensitive issues can lead to an emotional response, either cathartic or distressing (41). At the end of the interview there will be two questions regarding the experience of the cognitive assessments; this will provide information regarding the tolerability of performing such structured assessments in this patient group.

Statistical Analysis

Primary Endpoint:

To measure the change in mean HVLT-R scores between baseline and day 28 at the end of the first phase 1 cycle of treatment.

Secondary Endpoints:

To measure the mean change in COWA, CTMT, FACT-cog from baseline to day 8 and 28

To measure the mean change in HVLT_R from baseline to day 8

To measure the correlation between HADS and the Brief Fatigue Inventory and the HVLT_R, CTMT, and FACT-cog and COWA scores at baseline and other time-points, respectively.

All data from the cognitive function assessments, questionnaires and fatigue scores will be presented where appropriate as number of observations, means, standard deviations, medians and

For peer review only

interquartile range , respectively. The primary endpoint will be analysed in the first instance using a paired t-test if the data are normally distributed, if not, a non-parametric method such as the Wilcoxon matched pairs signed rank test will be used. Secondary endpoints in the first instance will be measured in the same way. Any repeated measures analyses will be carried out using ANOVA or Friedman's non-parametric test, respectively. Dependant on distribution being normal or otherwise spearman's rank or pearson's will be used to look at correlation between the objective measures. Regression analyses will be used to examine the effects of any confounding factors such as age, gender and co-morbidities on outcome. Where appropriate, data will be presented together with 95% confidence intervals.

Qualitative Data Analysis

The digital audio recording will be transcribed verbatim. The data will be organised using the Nvivo software package. The premise of thematic analysis is the examination and recognition of patterns within the data that are ultimately categorised into meaningful themes (42). Thematic analysis can use different approaches and be applied to varying types of data, it may be theory driven or content driven. In this instance it will be the content in the data that will drive the analysis (43).

Integration of data

At the stage of interpretation and reporting a mixed methods matrix will be utilised as a joint display of findings. This is a suggested practical way of viewing and analysing the different types of data together and offers a tool to guide the narrative during reporting (44,45). The comprehensive results and analysis will be held in a separate database. This matrix is to demonstrate findings in terms of any changes so that they can be considered during the interpretation stage in a holistic way taking into account both quantitative and qualitative findings.

Patient and Public Involvement

The protocol has had patient and public involvement in the design and development stage. The protocol framework was presented and discussed at a Royal Marsden Hospital Patient and Carer Research Panel, feedback led to some changes in the number of assessments. A patient representative was consulted on and helped to develop the questions for the semi-structured interview.

For peer review only

Cognitive Function Protocol for submission to BMJ

Ethics and dissemination

The study has obtained Research Ethics Committee approval in the UK reference number: 18/LO.0084 and full Health Research Authority approval IRAS project number: 235668. The study is currently open to recruitment after a long pause in recruitment due to the COVID-19 pandemic.

The literature review as the background work for the study was presented as a poster abstract at the International Cognition and Cancer Task Force conference in Sydney 2018. The plan will be to publish findings in a peer reviewed journal and at relevant conferences once study complete.

For peer review only

References

1) Wong. KM. Capasso. A. Eckhardt. SG. (2015) The changing landscape of phase I trials in oncology *Nature Reviews Oncology* (13) 106-117
2) Ivanova.A. (2007) Phase I trials in oncology *Wiley Encyclopedia of clinical trials* Wiley and sons.inc. Available at **http://onlinelibrary.wiley.com/doi/10.1002/9780471462422.eoct307/full** [Accessed on 26th July 2017]
3) National Cancer Institute (2010) Common Terminology Criteria for Adverse Events (CTCAE) Version 4 available at **https://evs.nci.nih.gov/ftp1/CTCAE/CTCAE_4.03_2010-06-14_QuickReference_5x7.pdf** [Accessed on 31st July]
4) Ng. T. Cheung. Y. Ng. Q. Ho. H and Chan. A. (2014) Vascular Endothelial Growth Factor Inhibitors and Cognitive Impairment: Evidence and Controversies. *Expert Opinion on Drug Safety* 13 (1) DOI: 10.1517/14740338.2013.82803
5) Selamat. M. Loh. S. Mackenzie. L. Vardy. J (2014) Chemobrain Experienced by Breast Cancer Survivors: A Meta-Ethnography Study Investigating Research and Care Implications *PLoS ONE* 9(9) Available at **http://journals.plos.org/plosone/article?id=10.1371/journal.pone.0108002 e108002** [Accessed on 15th April 2017]
6) Fitch M. Armstrong J. and Tsang S (2008**)** Patients' experiences with cognitive changes after chemotherapy *Cancer Oncology Nursing* 18(4): 180-92
7) Myers.C. and Perry.J. (2012) *Cognition and Cancer* Cambridge Medicine
8) Eysenck.M and Keane.M (2010) *Cognitive Psychology: A Student's Handbook.* Sixth Edition Psychology Press
9) Harvey PD. Domains of cognition and their assessment . *Dialogues Clin Neurosci*. 2019;21(3):227-
10) Ahles T. (2012) Brain Vulnerability to chemotherapy toxicities. *Psycho-oncology* 21(11): 1141-1148
11) Boykoff.N. Moieni. M and Subramanian.SK. (2009) Confronting chemobrain: an in-depth look at survivors' reports of impact on work, social networks and health care response. *Journal of Cancer Survivorships* 3 (4): 223-32
12) Fitch M. Armstrong J. and Tsang S (2008**)** Patients' experiences with cognitive changes after chemotherapy *Cancer Oncology Nursing* 18(4): 180-92

13) Martin. M. Clare. L. Altgassen. A. Cameron and M. Zehnder. F. (2011) Cognition based interventions for healthy older people and people with mild cognitive impairment *Cochrane Database of Systematic Reviews* (1) **http://www.healthevidence.org/view-article.aspx?a=cognition-based-interventions-healthy-older-people-people-mild-cognitive-21371** [Accessed on 10th May 2016]
14) Gerhring. K. Roukema. and JA. Sitskoorn. MM. (2012) Review of recent studies on interventions for cognitive deficits in patients with cancer. *Expert Reviews of Cancer Therapy* 12(2) 255-69
15) Ganz PA (2011) "Doctor will the treatment you are recommending cause chemobrain". *Journal of Clinical Oncology* 30(3):229-31
16) Deeks E (2015) Olaparib: first global approval *Drugs 75 (2): 231-40*
17) Moore. H and McSherry. W. (2013) Ethical Considerations in Translational Research. *Cancer Nursing* 12(10): 22-28
18) Wefel. J. Ahles. T. Vardy. J and Schagen. S (2011) International Cognition and Cancer Task Force recommendations to harmonise studies of cognitive function in patients with cancer *The Lancet Oncology* (12)7 703-708
19) Schilling V. Jenkins V and Morris R (2005**)** The effects of adjuvant chemotherapy for breast cancer-preliminary results of an observational study. *Breast* 14 (2): 142-150
20) Schagen SB. Muller MJ. Boogerd W. Mellenbergh GJ and Van Dam FS (2006) Change in cognitive function after chemotherapy: a prospective longitudinal study in breast cancer patients. *Journal of the National Cancer Institute* 98(23):1742-5.
21) Stewart A. Collins B. Mackenzie J. Tomiak E. Verma S and Bielajew C (2008) The cognitive effects of adjuvant chemotherapy in early stage breast cancer: a prospective study. *Psycho-oncology* 17(2):122-30.
22) Ahles.T. Saykin.A. McDonald.B. Li. Y. Furstenberg.C. Hanscom.B. Mulrooney. T . Schwarrtz. G and Kaufman.P (2010) Longitudinal Assessment of Cognitive Changes Associated With Adjuvant Treatment for Breast Cancer: Impact of Age and Cognitive Reserve. *Journal of Clinical Oncology* 28 (29): 4434-4440
23) Collins B. Mackenzie J. Tasca G. Scherling C and Smith A (2012**)** Cognitive Effects of chemotherapy in breast cancer patients a dose-response study. *Psycho-Oncology* 22(7): 1517-1527
24) Collins B. Mackenzie J. Tasca G. Scherling. C. and Smith. A.(2013) Persistent Cognitive Changes in Breast Cancer Patients 1 Year Following Completion of Chemotherapy. *Journal of the International Neuropsychological Society (20*):1-10
25) Jones D. Vichaya E. Wang X. Sailors M. Cleeland C and Wefel. J (2013) Acute Cognitive Impairment in Patients With Multiple Myeloma Undergoing Autologous Haematopoietic Stem Cell Transplant *Cancer* 119(23):4188-95
26) Cruzado J. Lopez- Santiago S. Martinez-Marin V. Jose-Moreno G. Custadio AB and Feliu J (2014) Longitudinal Study of cognitive dysfunctions induced by adjuvant chemotherapy in colon cancer patients. *Supportive Cancer Care* 22(7): 1815-1823
27) Kohli S. Griggs J. Roscoe J. Pascal JP. Bole C. Mustian K. Hill R. Smith K. Gross H and Morrow. G. (2007) Self-Reported Cognitive Impairments in Patients with Cancer. *Journal of Oncology Practice* 3(2):54-9
28) Quesnel. C. Savard.J.and Ivers.H.(2009) Cognitive impairments associated with breast cancer treatments: results from a longitudinal study. *Breast cancer research and treatment (*116) 113-123
29) Klaver, K.M., Duijts, S.F.A., Engelhardt, E.G. *et al.* Cancer-related cognitive problems at work: experiences of survivors and professionals. *J Cancer Survivorship* **14,** 168–178 (2020). https://doi.org/10.1007/s11764-019-00830-5

For peer review only - http://bmjopen.bmj.com/site/about/guidelines.xhtml

30) Bendall. JC. Rodon. J. Burris. HA (2012) Phase I dose escalation study of BKM120 an oral pan-class PI3K inhibitor in patients with advanced solid tumours Journal of Clinical Oncology (30) -282-290
31) Fong P. Boss D. Yap T. Tutt A. Peijun W. Mergei-Roelvink M. Mortimer P. Swaisland H. Lau A. O'Conner M. Ashwoth A. Carmichael J. Kaye S. Schellens J and De-Bono. J (2009*)* Inhibition of Poly (ADP Ribose) Polymerase in Tumours from BRCA Mutation carriers. *The New England Journal of Medicine* 361(2):123-134
32) Mckay. R. Velasco. D. Werner. L. Bellmunt. J. Harshman. L. Swenney. C. Rosenberg. J. Hirsch. M. Signoretti. S. Van Allen. E. Walsh. M. Vaishampayan. U and McDermott. D (2014) A Phase I study of buparlisib and bevacuzimab in patients with metastatic renal cell carcinoma progressing on vascular endothelial growth factor targeted therapies *Cancer* (122) (15)
33) Heinrick. M. Jones.R. Von-Mehren. M. Schoffski.P Bauer. S. Mir.O. Cassier. P. Eskens. F. Shi. H.Alvarez-Diez. T. Schmidt-Kittler.O. Healey.M. Wolf.B. George. S (2017) *Clinical Activity of BLU-285 in advanced gastro-intestinal stromal tumours* (GIST)Journal of Clinical Oncology 35 DOI:10.1200/JCO.2017.35.15-suppl.11011
34) Upadhaya. N. and Guragain. S. (2014) Comparison of cognitive functions between male and female medical students: A Pilot study Journal *of Clinical and Diagnostic Research* 8 (6)
35) Benton A, Hamsher K and Sivan. A (1978) Multilingual Aphasia Examination Manual of Instructions 3rd Edition Psychological Assessment Resources
36) Brandt. J. and Benedict.R. (2001) Hopkins Verbal Learning Test-Revised- Professional Manual Psychological Assessment Resources
37) Reynolds C (2002) Comprehensive Trail-Making Test Examiners Manual Pro-Ed
38) FACIT.org (2017) Available from http://www.facit.org/FACITOrg/Questionnaires
39) Stern. F (2014)The Hospital Anxiety and Depression Scale, Occupational Medicine, Volume 64, Issue 5 Pages 393–394, https://doi.org/10.1093/oc
40) MD Anderson Cancer Centre (1995) Brief Fatigue Inventory Symptom Assessment Tool http://www.mdanderson.org/education-and-research/departments-programs-and-labs/departments-and-divisions/symptom-research/symptom-assessment-tools/brief-fatigue-inventory-bfi.html cmed/kqu024
41) Johnson.B and Clarke. J (2003) Collecting sensitive data: the impact on researchers *Qualitative Health Research* 13(3): 421-34
42) Guest. G. (2012) *Applied Thematic Analysis* Thousand Oaks California
43) Braun V and Clarke V (2006) Using thematic analysis in psychology. *Qualitative Research in Psychology* 3(2): 77-101
44) Johnson R. Onwuegbuzie A and Turner. L. (2007) Toward a definition of mixed methods research. *Journal of mixed methods* 1(2): 112-133

Author's Contributions

Sarah Stapleton – Primary Researcher, Research design concept, Protocol Development and Writing

Prof. Anne-Sophie Darlington- PhD primary supervisor, Protocol Development

Prof. Johann De-Bono – Clinical oversight of the study as head of unit, review of protocol development

Prof. Theresa Wiseman – Principal Investigator , PhD supervisor, Protocol development ,

Cognitive Function Protocol for submission to BMJ

Competing Interests

Sarah Stapleton None declared however SS post is funded by Cancer Research UK

Prof. Anne-Sophie Darlington None declared

Prof. Theresa Wiseman None declared

Prof. Johann De-Bono: JDB has served on advisory boards and received fees from many companies including Astra Zeneca, Astellas, Bayer, Bioxcel Therapeutics, Boehringer Ingelheim, Cellcentric, Daiichi, Eisai, Genentech/Roche, Genmab, GSK, Janssen, Merck Serono, Merck Sharp & Dohme, Menarini/Silicon Biosystems, Orion, Pfizer, Qiagen, Sanofi Aventis, Sierra Oncology, Taiho, Vertex Pharmaceuticals. He is an employee of The ICR, which have received funding or other support for his research work from AZ, Astellas, Bayer, Cellcentric, Daiichi, Genentech, Genmab, GSK, Janssen, Merck Serono, MSD, Menarini/Silicon Biosystems, Orion, Sanofi Aventis, Sierra Oncology, Taiho, , Pfizer, Vertex, and which has a commercial interest in abiraterone, PARP inhibition in DNA repair defective cancers and PI3K/AKT pathway inhibitors (no personal income). He was named as an inventor, with no financial interest, for patent 8,822,438. JDB is a National Institute for Health Research (NIHR) Senior Investigator. The views expressed in this article are those of the author(s) and not necessarily those of the NHS, the NIHR, or the Department of Health.

Funding Statement

The research received no specific grant from any funding agency in the public, commercial or not for profit sectors. However the primary researcher's post is funded by Cancer Research UK.

Acknowledgements

The author would like to acknowledge the Royal Marsden Patient and Carer Research Review Panel for their contribution to the protocol development.

-

For peer review only

Figure 1

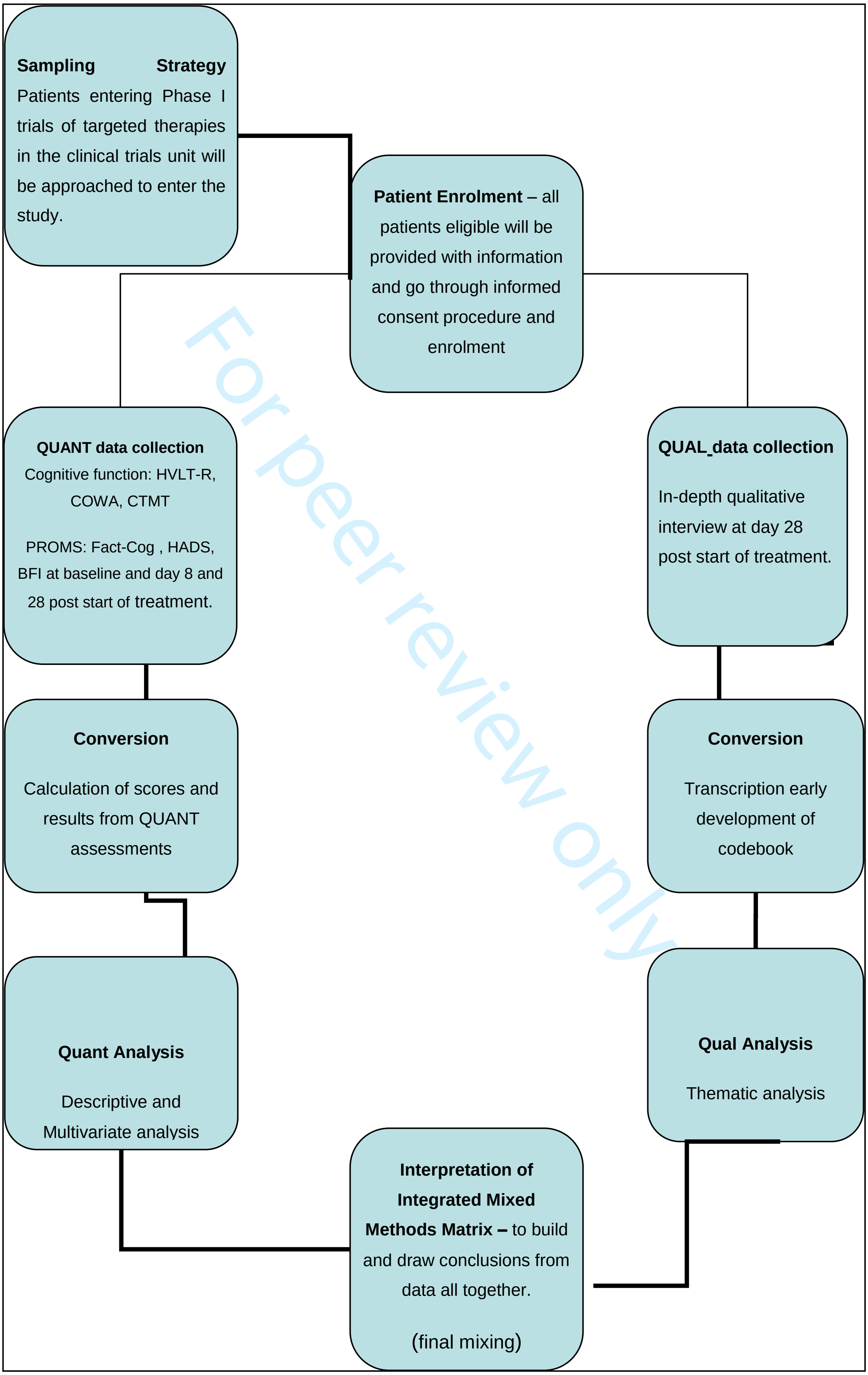

For peer review only

Figure 2

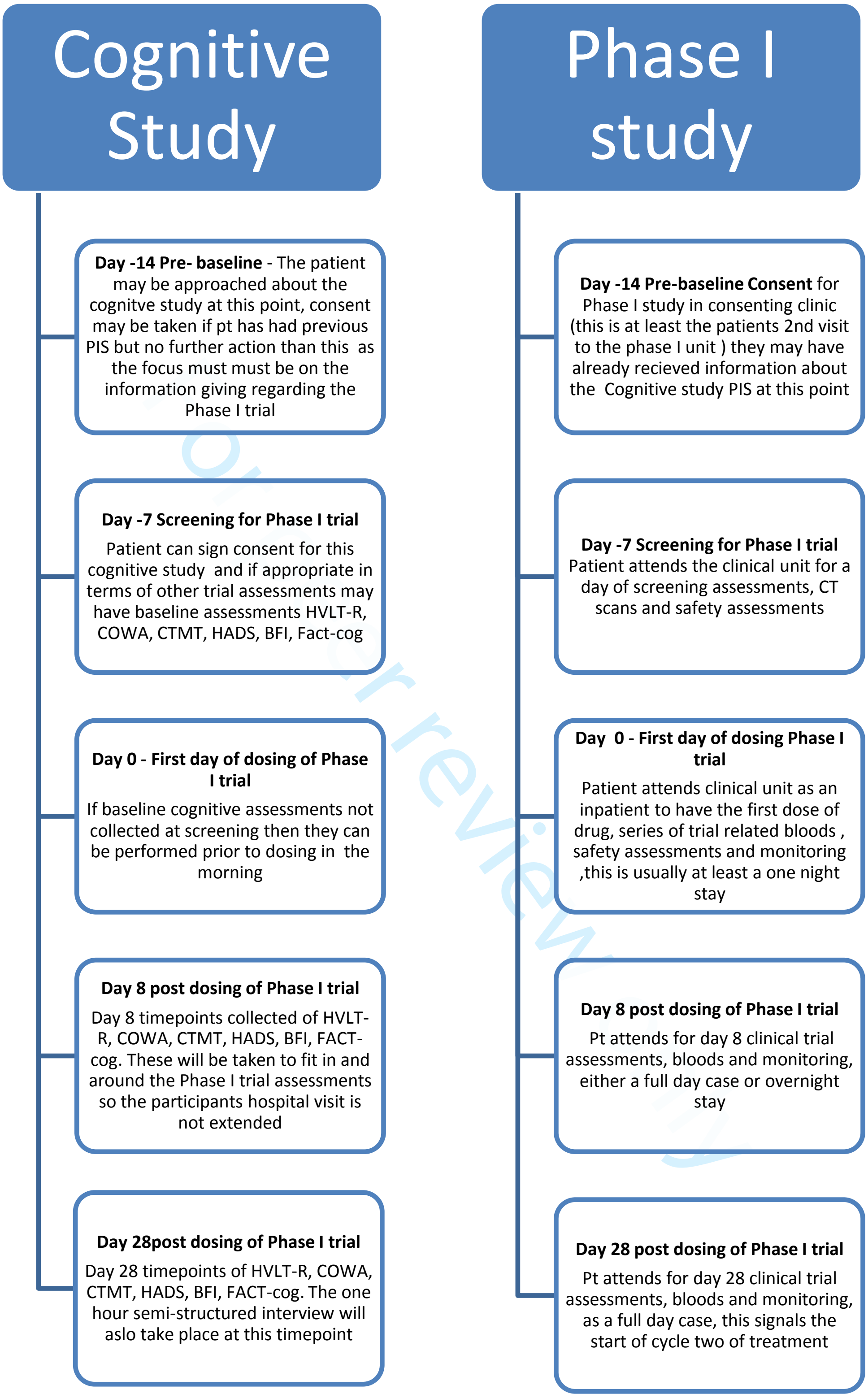

For peer review only - http://bmjopen.bmj.com/site/about/guidelines.xhtml

Table 1 **Research sub Questions aligned with methods of data collection and analysis**

Sub Question (SQ)	Methods of Data Collection	Analysis
SQ1: Are targeted therapies given within the context of Phase I trials associated	Cognitive assessments using comprehensive standardised cognitive assessment tools.	Descriptive analysis of number of patients with deterioration on cognitive function.
SQ2: What is the experience of cognitive function and the impact of any deterioration on the lives of these people on Phase I trials?	Individual in-depth interviews and patient reported outcomes.	Thematic analysis and descriptive stats
RQ3: How acceptable is the process of standardised cognitive assessments alongside the Phase I clinical trial?	Attrition rates recorded and Qualitative enquiry as part of the interview to ask how acceptable the cognitive assessment battery has been	Presented as percentages with reason for attrition recorded.
RQ4: How accurate are nurse performed cognitive assessments (the nurse has accredited training in Psychometric testing and is being supervised by a clinical psychologist).	Measurement of number of approved accurate cognitive assessments undertaken as validated by psychologist.	Percentages of positive validation by supervising Psychologist.

For peer review only

Table 2

**Validated Cognitive Assessment Tests**	**Process**	**Time taken to complete test**
Controlled Oral Word Association **COWA** (35)	This is an oral fluency test. The assessment is made by asking the participant to make verbal associations to letters of the alphabet by saying as many words one can think beginning with a given letter. A sheet is available to record verbatim response. Total number of acceptable responses provides the raw score.	5-10 minutes
Hopkins Verbal Learning Test _Revised **HVLT_R (36)**	This is a word list learning and memory test. Each form of the test consists of 12 nouns with 4 items taken from each semantic category. The word list is read to the patient who then has to recall as many words as possible. The task is repeated two or three times. A 25 minute interval is given after which a delayed recall test is performed. The tests are then scored in terms of total recall, delayed recall and retention %.	40 mins (including 25 minutes for delayed recall test)
Comprehensive Trail Making Test **CTMT (37)**	This test measures executive function, concentration and task switching. The test consists of a standardised set of visual search and sequencing tests. The raw score for each trail test is the number of seconds required for the participant to complete; time is added per error as the examiner corrects them.	5-12 minutes
**Patient Reported Outcomes**	**Process**	**Time taken to complete test**
**Fact-Cog** (38)	This is a 35 item questionnaire measuring the patient's own perception of cognitive function changes. It is based on a 5 point rating scale "in the last 7 days timeframe".	15 minutes
Hospital Anxiety and Depression scale **HADS (39)**	The hospital anxiety and depression score is a 14 item scale for patients who may have other medical problems. It is essentially a screening tool for anxiety and depression. 7 items cover anxiety and 7 items cover depression. Each item has score of 0-3 making a potential score between 0-21 for each problem.	15 mins
Brief Fatigue Inventory **BFI (40)**	The brief fatigue inventory is a six item assessment tool measuring the patients' levels of fatigue. It records changes over short periods with a focus on fatigue in the previous 24 hours.	5 mins
**Qualitative data collection**	**Process**	**Time taken to complete interview**
Semi-structured interviews	The patient participates in a one hour semi-structured interview. This takes place on the same day as the final objective measures. The questions explore the experience of any perceived cognitive issues as well as coping strategies and any other factors that may influence this experience. Within this interview there are is a short section asking questions regarding acceptability of the objective measures as part of the feasibility aspect of this study.	One hour

For peer review only

BMJ Open

The Impact of targeted therapies given within Phase I trials on the cognitive function of patients with advanced cancer.

Journal:	*BMJ Open*
Manuscript ID	bmjopen-2021-050590.R1
Article Type:	Protocol
Date Submitted by the Author:	07-Feb-2022
Complete List of Authors:	Stapleton, Sarah ; Royal Marsden Hospital Sutton, Drug Development Unit ; University of Southampton, Faculty of Health Sciences<br>Darlington, Anne-Sophie; University of Southamptom, Faculty of Health Sciences<br>de Bono, J. S.; Royal Marsden NHS Fdn Trust; Institute of Cancer Research Division of Cancer Therapeutics<br>Wiseman, Theresa; The Royal Marsden NHS Foundation Trust, ; University of Southampton, Faculty of Health Sciences
**Primary Subject Heading**:	Oncology
Secondary Subject Heading:	Pharmacology and therapeutics
Keywords:	CLINICAL PHARMACOLOGY, Adult oncology < ONCOLOGY, Delirium & cognitive disorders < PSYCHIATRY, Clinical trials < THERAPEUTICS

SCHOLARONE™
Manuscripts

Cognitive Function Protocol for submission to BMJ

Title: The Impact of targeted therapies given within Phase I trials on the cognitive function of patients with advanced cancer.

Short Title: Cognitive Function and Phase I cancer drug trials

Authors:
Sarah. E. Stapleton (1) Prof. Anne-Sophie Darlington (2) Prof. Johann De-Bono (1,3) Prof.Theresa Wiseman (1)

1 Royal Marsden Hospital Downs Rd Sutton Surrey Sm2 5PT

2 University of Southampton University Rd Southampton SO17 1BJ

3 Institute of Cancer Research 15 Cotswold Rd Sutton SM2 5NG

Corresponding Author: Sarah Stapleton Royal Marsden Hospital Downs Rd Sutton Surrey SM2 5PT sarah.stapleton@rmh.nhs.uk

Keywords: Drug Development , Early Phase I Clinical Trials, Cognitive function, Cognitive impairment, Cognitive assessment

For peer review only - http://bmjopen.bmj.com/site/about/guidelines.xhtml

Cognitive Function Protocol for submission to BMJ

Abstract

Introduction: Novel therapies such as small protein molecule inhibitors and immunotherapies are tested in early phase trials before moving to later phase trials and ultimately standard practice. A key aim of these clinical trials is to define a toxicity profile, however the emphasis is often on safety with measurements of organ toxicity. Other subjective side effects can be underreported because they are not measured formally within the trial protocols. The concern from clinical practice is that cognitive toxicity is poorly studied and may be under-reported in this context. This could lead to toxicity profiles treatments not being fully described and patients with unmet need in terms of acknowledgement and support of symptoms. This protocol outlines a framework of an exploratory study with feasibility aspects to investigate the impact and experience of cognitive changes for patients on Phase I trials.

Methods and analysis: This is a mixed methods study combining quantitative and qualitative approaches. 30 Patients with advanced cancer who are participating in Phase I trials of novel targeted therapies in the early clinical trials unit of a specialist cancer centre. A test battery of validated cognitive assessments will be taken alongside patient reported outcome measures at three time-points from baseline, day eight and day 28 post start of treatment. At day 28 a semi structured interview will be conducted and the narrative thematically analysed. Results will be integrated to offer a comprehensive description of cognitive function for in this patient group.

Ethics and dissemination: The study has received full HRA and ethical approval. It is the first study to introduce formal cognitive assessments in a cancer Phase I trial context. The study has to potential to highlight previously unreported side effects and more importantly unmet need in terms of care for our patients who are participating in the trials.

For peer review only

Strengths and Limitations

- This is the first study to investigate cognitive function longitudinally during the course of Phase I trials.
- The findings of the trial will establish feasibility of conducting more formal and consistent cognitive testing in early clinical drug trial protocols.
- Describing the symptoms of patients in this group will help to identify unmet need in terms of support and management.
- Recommendations of the International Cancer and Cognition Task Force to harmonise research in this field were adopted in the trial design.
- Due to the exploratory nature and feasibility aspects of the study, the sample size is not statistically powered. A future study would benefit from a powered calculated sample size to demonstrate any treatment related change with more certainty.

Cognitive Function Protocol for submission to BMJ

Introduction

In cancer drug development advances in the understanding of genetics and the biochemical pathways involved in cancer have helped researchers to rationally design “molecularly targeted drugs”. These novel targeted therapies and immunotherapies are first tested clinically in patients within Phase I trials in cancer centres. Due to the experimental nature of Phase I trials and the fact that little is known about the toxicities, patients need to have exhausted all standard treatment options to participate. To contextualise, these patients will generally have advanced cancer, pre-treated disease and often a limited prognosis. It is a heterogenous patient population in terms of tumour types. However, what the patients do have in common is a shared experience of the continuous cycle of treatment and disease progression to the point where there is no further treatment available. Patients on Phase I trials often have significant tumour burden with metastatic disease and a life limiting prognosis. These patients remain motivated to participate in Phase I trials of experimental therapies and despite having advanced progressive disease the criteria for participating in Phase I means the patients do have to have a certain level of fitness to meet eligibility requirements of specific protocols.

At this stage of drug development, the main aims are to find the optimal dose, investigate the pharmacology and to define the toxicity profiles (1). When considering toxicity profiles of these potential new treatments the emphasis is on safety and this is reflected in many of the standard assessments in the protocols such as cardiology monitoring and renal and liver function tests (2). Clinicians and nurses involved in the conduct of Phase I trials do perform medical history taking and nursing assessments to gain a full perspective of other side effects reported but they are interpreted and recorded in a subjective manner usually using the Common Terminology Criteria for Adverse Events (CTCAE) which is a National Institutes of Health – National Cancer Institute, Division of Cancer Treatment and Diagnosis, Cancer Therapy Evaluation Program initiative. It is a multi-dimensional tool designed specifically for use in clinical trials. The tool enables the clinician to categorise symptoms that are graded 0-5, translating respectively to none, mild, moderate, severe, life threatening and death. The use of the CTCAE is commonplace in all Phase I trials (3).

This exploratory study investigates the impact of targeted therapies within the context of an experimental trial on the cognitive function of patients with advanced cancer. It is suspected that some of the targeted therapies tested within Phase I trials may have a negative impact on the patient’s cognitive function. Evidence from clinical practice suggests that patients are experiencing symptoms relating to cognitive function, however this does not seem to translate to reports of cognitive toxicity within clinical trial data. As cognitive function is not formally assessed the extent, nature or domain of

For peer review only - http://bmjopen.bmj.com/site/about/guidelines.xhtml

cognitive impairment is not well described. There is also suggestion in literature that the difficulty in ascertaining the relationship between cognitive impairment and targeted therapies may be due to the underreporting of cognitive symptoms in clinical trials (4,5).

Rationale

Impairment of cognitive function in the field of traditional cytotoxic chemotherapy has been investigated and it is known that such a symptom impairs quality of life (6). Therefore, if cognitive function was investigated and described in the context of patients on Phase I trials, it could provide a basis of knowledge on which to develop interventions and coping strategies with the aim of preventing or ameliorating the effects of this possible decline in cognitive function.

Cognitive function is described as the internal processes involved in making sense of the world and the capacity to make decisions (7). Such processes include attention, perception, learning, memory, language, problem solving, reasoning and thinking (8). These functions are defined in terms of domains that can broadly be divided into sensory and motor skills, memory, attention and concentration, executive function (which includes reasoning and problem solving) and language and verbal skills (9). Whilst the domains overlap in terms of function each can be assessed using validated cognitive assessment tools. Impaired cognitive function in cancer patients can present as loss of memory, difficulty with word finding, loss of concentration and difficulty in task switching (10). Even though cognitive function is not assessed as part of the Phase I trials, anecdotal reports from some patients suggest that they experience some deterioration whilst on the trial often temporal to dosing of drug.

For patients this has implications in that they may have a symptom they feel unable to discuss. This in turn can mean that they do not receive any help or support regarding the problem and can be isolating and frightening for the patient (11). Not having the symptom acknowledged or having no information to warn about the possibility of it happening has been reported to be one of the most distressing and difficult things for patients to cope with (11,12).

Implications on a broader scale will mean that better defined toxicity profiles will improve understanding of consequences of treatments. This will assist in developing interventions that help patients make informed choices about having certain treatments and be better equipped to cope with the consequences of them. This may be as simple as recognition of the potential problem with

For peer review only - http://bmjopen.bmj.com/site/about/guidelines.xhtml

information and preparation for the patient or the implementation of strategies to help with symptoms such as compensatory memory techniques, rehabilitation and relaxation (13,14,).

In terms of relevance the numbers of patients initially receiving such treatments in Phase I trials may be relatively small, usually tens to hundreds. Nevertheless for these patients with a limited prognosis issues regarding side effects are important. Once a drug has gone through the phases of testing and is licensed for practice the numbers of patients receiving these treatments could then become thousands (15). The number of cancer patients participating in clinical trials is increasing (16). Therefore it is important to find out accurately what is happening to patients on clinical trials so they can be supported with the management of side effects such as diminished cognitive function. In order to begin to address this and improve the care and understanding of patients on Phase I trials this proposed study will be the first exploration using formal assessment of cognitive function in this context.

Evidence Gap

Early work in chemotherapy related cognitive impairment highlighted a disparity in trial designs and some limitations in research in this field such as the use of cross-sectional trial design offering no baseline from which to measure change. Lack of consistency in the type of cognitive assessment methods used in the trials also lead to broad differences in reporting of the prevalence of cognitive impairment. This led to a group of expert cognitive researchers founding an international multidisciplinary group known as the International Cancer and Cognition Task Force (ICCTF). The ICCTF was founded in 2004 and one of the aims was to produce guidelines to harmonise study design. The recommendations were to include pre-treatment assessment, longitudinal follow up, the use of a control group and a core set of cognitive assessment tests when investigating cognitive function in cancer patients (17). All recommendations will be adopted for this protocol except the use of a control group. It would be difficult to match a control for this patient group as the main reason that patients do not enter a Phase I trial is due to a deterioration in clinical condition. Therefore patients in a comparable situation would likely be receiving palliative care only and it may not be appropriate to ask them to participate in order to provide a control.

The subsequent studies that followed these guidelines continued to show associations between systemic chemotherapy treatment and a decline in cognitive function as well as discovering that patients with cancer often have a degree of cognitive impairment at baseline when compared to matched healthy controls. (18 -20). The studies also demonstrated the manifestation of cognitive

For peer review only - http://bmjopen.bmj.com/site/about/guidelines.xhtml

deficits which presented as loss of concentration, memory, verbal fluency and executive functioning which includes higher order cognitive abilities of planning and abstract thinking. Due to the longitudinal design some have been able to demonstrate compelling evidence of dose dependent relationships between treatment and severity of diminished cognitive function (21-25).

Some of the studies also highlighted the value of collecting the patient's own perception of cognitive function as they showed that this can vary from the validated cognitive assessment measures, (26,27). What is not addressed in many of these studies is the meaning to the patient in terms of how it may affect their lives. Just a small number of qualitative studies have been conducted exploring the impact of cognitive function on the quality of life of patients on traditional cytotoxic chemotherapy. They show that there is a negative effect on work, sometimes ability to return to previous occupations and effects on emotional, social and psychological aspects of daily life (28)

A scoping literature review was conducted with the aim of understanding the current status of assessment and reports of cognitive function within Phase I trials. The scoping review utilized the Preferred Reporting Items for Systematic reviews and Meta-Analyses extension for Scoping Reviews (PRISMA-ScR) Checklist as a framework (29). Papers were included if they reported cognitive symptoms or symptoms categorised as cognitive within the toxicity reports of the trial. The purpose of this was to identify any issues of relating to cognitive function including how it was assessed and reported. Also included were any studies investigating cognitive function in a Phase I population. Other observational studies that explored symptom presentation and reporting in Phase I trials were included. The purpose of this was to understand any factors that are specific to this patient population and context that may influence accurate identification of cognitive symptoms. Excluded were studies that did not include cognitive issues in the reports. Studies of patients with central nervous system tumours and paediatric patients were excluded as due to the nature of the disease and treatment there are cognitive issues specific to each of these patient's groups. Search limits included papers in English and dates of publication 2010-2021.

Just one study in the review specifically investigated cognitive function in a population of Phase I patients. The aim was to identify the relationship between cognitive function and intact decisional capacity in relation to informed consent. The cross-sectional design assessed the cognitive function of patients at baseline prior to starting the trial so there was no longitudinal assessment or feasibility assessment throughout the course of the clinical trial. The sample was representative of a Phase I population, outcome measures were valid, and associations of confounding variable were adjusted

For peer review only - http://bmjopen.bmj.com/site/about/guidelines.xhtml

for in the analyses. The research team found that a proportion of patients had cognitive impairment at baseline and those with worse cognitive function were less likely to understand and remember some of the key principles of the trial. The study was limited due to the cross-sectional design and the researchers do propose that further longitudinal work assessing cognitive function in patients on Phase I trials would be of value (30).

Evidence of cognitive toxicity reports in the Phase I trials was limited. A small number did report cognitive toxicity. The symptoms were not well characterised and sometimes aggregated or reported as a cognitive category rather than the symptom, making it difficult to understand exactly what the adverse events were.

A phase 1 trial investigating safety and efficacy of BLU-285, a highly selective targeting inhibitor of mutant KIT and PDGFR in a population of 116 patients with Gastro-Intestinal Stromal Tumour reported cognitive impairment as a toxicity. The symptoms are aggregated in the report as cognitive effects. The paper reports that 46% (n=115) have all grade cognitive effects and group together memory impairment, confusional state, cognitive disorder and encephalopathy within this category (31). This perhaps represents a lack of clarity in working definitions of cognitive impairments.

Other reports of cognitive toxicity within Phase I include a combination study investigating the maximum tolerated dose and toxicity profile of VEGF inhibitor and Pi3K inhibitor for patients did not include cognitive assessments but did include specific mental health diagnostic tools in the trial design. Despite the lack of cognitive assessments 10 %(n=30) of patients on the trial reported cognitive disturbance. This is another example of the symptom being reported as a category rather than the actual symptom, hence the presentation of the cognitive disturbance is impossible to elucidate. This vague description leads to a poor understanding of the symptom which is attributed as a severe side effect of treatments. Indeed the cognitive disturbance was graded severe enough to be classified as a dose limiting toxicity. A dose limiting toxicity is a drug related adverse event pre-defined in the protocol and is significant as it may prevent further dose escalation. This means that the recommended doses could be affected. (32).

Despite not including any cognitive assessments within the trial protocol, a Phase I trial of a Pi3K and mTor inhibitor in combination also reported" cognitive disorders " in some patients on the trial this is further categorised as memory loss, confusional state, attention deficit and agitation and causality was attributed to the investigational medicinal product (33). Because no formal cognitive assessments were incorporated into the study design, the severity and duration of the memory and attention loss is unclear.

For peer review only - http://bmjopen.bmj.com/site/about/guidelines.xhtml

None of the papers filtered in the review used formal cognitive assessments within the trial protocol, so the review confirms that there are no formal or consistent cognitive assessments as part of current standard practice within early clinical trials.

More recently studies are emerging that look at Patient Reported Outcome Measures (PROM) in Phase I trials, in particular the Patient reported outcomes version of the CTCAE (PRO-CTCAE) which is a multidimensional PROM specifically designed by the NCI to capture symptomatic adverse events in clinical trials (34). Whilst its use is commonplace in Phase 2 and 3 trials it's used less in early Phase I trials where definition of toxicity is a key endpoint (35). One study recruited 265 patients on Phase I trials and administered the PROM at three timepoints over the course of the Phase I trial. In the case of cognitive events there are two items on the scale of memory impairment and impaired concentration. The study team describe a difference between patients reports and clinician reports as 25.9% of patient reports of memory as compared to 0.5% of clinicians and in the case of impaired concentration 0.7 to 28.1 % respectively. A similar study using the MD Anderson using the MDAnderson symptom inventory (MDASI) which is a also patient reported questionnaire focusing on 13 core common cancer symptoms (36). It has one item relating to cognitive function labelled as "difficulty remembering". This study showed that up to 41% of patients identified as having difficulty remembering.

The number of patient reported experiences of cognitive impairment in the PROM's studies are incongruous with the many papers filtered in this review where there are simply no or very limited reports of cognitive impairment. This highlights potential unmet need in this patient group.

In summary, there is a lack of evidence to show the effects on cognitive function for patients on early Phase I clinical trials. This has a two-fold consequence. Firstly that the side effects of the drugs may not be as well characterized and may only become apparent in latter stages of drug development or when a drug becomes standard practice. Secondly patients who are participating in Phase I trials and may have unmet need in terms of identification and support of symptoms of cognitive impairment. This research protocol outlines an exploratory framework for a study utilizing mixed methods to both identify the existence of cognitive decline in these patients but also describe the impact this has on their everyday lives.

For peer review only - http://bmjopen.bmj.com/site/about/guidelines.xhtml

3. Research Question and Aims

The study is not hypothesis testing and is framed as an exploratory study. The overarching aim is to identify, characterise and gain an understanding of the effects on the cognitive function of patients on novel targeted therapies given within the context of Phase I trial. The study has an added feasibility aspect to gain an understanding of how acceptable and appropriate nurse led cognitive assessments are in this patient group due to the existing potential burden of the Phase I trial commitments. Therefore the overarching research question for the study is:

“What is the impact of the targeted therapies given within the context of a Phase I trial on the cognitive function of patients with advanced cancer?”

The research question to explore this can be broken down into six sub questions. The research sub questions are presented in a Table 1 below aligned with the objectives, methods and analysis.

For peer review only - http://bmjopen.bmj.com/site/about/guidelines.xhtml

Table 1 Research question aligned with objective, methods and analysis

**Research Question (RQ))**	**Objectives**	**Methods of Data Collection**	**Analysis**
RQ1 : What is the cognitive function of the patients on Phase I trials of novel therapies	To describe the cognitive function over time of this patient group	Cognitive assessments of memory (Hopkins Verbal Learning Test-r) executive function (Comprehensive Trail Making Test A and B) and verbal fluency (Controlled Oral Word Association Test)	Age adjusted T scores will be presented as means and standard deviations with confidence intervals for each timepoint. Proportions of patients with CI will be presented at each timepoint.<br><br>ANOVA repeated measures will be conducted to describe the change over the three timepoints.
RQ2: What is the experience of cognitive function and the impact of any deterioration on the lives of these people on Phase I trials?	To explore and understand the patient narrative surrounding cognitive function in this context.	Individual in-depth interviews<br><br>PROM' of perceived CI Fact-cog	Thematic analysis (Braun and Clark)<br><br>Decline at each timepoint will be present as predefined Minimum Clinically Important Differences (Cut off points are available for perceived impairment subscale that can be used to present proportions of patients with impairment at each timepoint).
RQ3: Do patient's perspectives mirror cognitive outcome measures overtime?	To compare, contrast and look for confirmation, divergence and an in-depth characterization of cognitive function in this patient group	Combined qual and quant	Integration at analysis stage using mixed methods matrix to interpret findings
RQ4 What influence do potential confounding factors of depression, anxiety and fatigue have on the cognitive scores in this patient group?	To assess the impact of potential confounding variables on cognitive decline in this patient group.	PROM's fatigue, anxiety and depression	A multiple regression analyses will be conducted to examine the influence of the confounding variables
RQ5: How acceptable are the cognitive assessments alongside the Phase I clinical trial?	To measure acceptability of extra cognitive assessments alongside Phase I trials.	Attrition rates recorded and<br>Qualitative enquiry as part of the interview to ask how acceptable the cognitive assessment battery has been	Measurements of uptake of trial, refusal to enter study (with reasons if possible) attrition rates (presented as numbers and percentages)<br>Frequency of positive responses in qualitative questions<br>Coding and thematic analysis of narrative response to enquiry about acceptability of questions.
RQ6: How accurate are nurse led cognitive assessments (the nurse has accredited training in Psychometric testing and is being supervised by a clinical psychologist).		Measurement of number of approved accurate cognitive assessments undertaken as validated by psychologist.	Percentages of positive validation by supervising Psychologist.

For peer review only

Methods and Analysis

This is a mixed methods study design using both qualitative and quantitative approaches with equal weighting to gain a comprehensive description of cognitive function for patients with advanced cancer on a Phase I trial of a novel therapy.

Sample

A non-probability sampling strategy technique of convenience sampling will be used. Most of the patients entering a Phase I trial will be eligible for this study therefore this sampling technique will allow for the quickest access to patient recruitment. The patients will be recruited from the Phase I Clinical Trials Unit within a specialist cancer hospital. The patients attend the unit on a weekly basis. The patient group is heterogeneous in terms of tumour type, age and gender. However the similarities within the sample population will be the stage of disease, the types of previous treatment and context. Although a convenience sample will be used there will be some stratification in terms of gender. Due to sample size any stratification of other possible confounding factors such as age, educational status will not be possible. Age and educational status (as a key component of cognitive reserve) have been shown to have an influence of altered cognitive function. The sample split between male and female will aim to be equal as it is known that there are cognitive differences relating to gender (37). As this is an exploratory study with feasibility aspects, the sample size will be 30 patients.

Inclusion Criteria

- Patients with a confirmed diagnosis of cancer
- Patients who are eligible for the trial and meet the trial specific eligibility criteria
- Patients who are allocated to and commence a Phase I trial (either IV or oral)
- Patients who are over eighteen

Exclusion Criteria

- Patients with a primary central nervous system tumour
- Patients who have received whole brain radiotherapy
- Patients who are non-English speaking
- Patients with uncontrolled psychiatric disorders

For peer review only - http://bmjopen.bmj.com/site/about/guidelines.xhtml

Study Design

This is a mixed methods, longitudinal design study. The methods and schedule are as follows.

Insert Figure 1 Patient pathway aligned with Phase I trial

Quantitative Methods

This study follows the ICCTF recommendations of a core battery of validated cognitive assessments of memory, verbal fluency and executive functioning at baseline , day eight post start of trial treatment and day 28 post start of trial treatment. As well as the cognitive assessments that will be conducted by the primary researcher there will be patient reported outcome measures of fatigue , depression and anxiety and self- perception of cognitive function. Table 2: methods of data collection presents the data collection tools and how they are used.

Table 2 Methods of data collection

**Validated Cognitive Assessment Tests**	**Process**	**Time taken to complete test**
Controlled Oral Word Association **COWA** (35)	This is an oral fluency test. The assessment is made by asking the participant to make verbal associations to letters of the alphabet by saying as many words one can think beginning with a given letter. A sheet is available to record verbatim response. Total number of acceptable responses provides the raw score.	5-10 minutes
Hopkins Verbal Learning Test _Revised **HVLT_R (36)**	This is a word list learning and memory test. Each form of the test consists of 12 nouns with 4 items taken from each semantic category. The word list is read to the patient who then has to recall as many words as possible. The task is repeated two or three times. A 25 minute interval is given after which a delayed recall test is performed. The tests are then scored in terms of total recall, delayed recall and retention %.	40 mins (including 25 minutes for delayed recall test)
Comprehensive Trail Making Test **CTMT (37)**	This test measures executive function, concentration and task switching. The test consists of a standardised set of visual search and sequencing tests. The raw score for each trail test is the number of seconds required for the participant to complete; time is added per error as the examiner corrects them.	5-12 minutes
**Patient Reported Outcomes**	**Process**	**Time taken to complete test**
**Fact-Cog** (38)	This is a 35 item questionnaire measuring the patient's own perception of cognitive function changes. It is based on a 5 point rating scale "in the last 7 days timeframe".	15 minutes
Hospital Anxiety and Depression scale **HADS (39)**	The hospital anxiety and depression score is a 14 item scale for patients who may have other medical problems. It is essentially a screening tool for anxiety and depression. 7 items cover anxiety and 7 items cover depression. Each item has score of 0-3 making a potential score between 0-21 for each problem.	15 mins
Brief Fatigue Inventory **BFI (40)**	The brief fatigue inventory is a six item assessment tool measuring the patients' levels of fatigue. It records changes over short periods with a focus on fatigue in the previous 24 hours.	5 mins

For peer review only - http://bmjopen.bmj.com/site/about/guidelines.xhtml

Qualitative data collection	Process	Time taken to complete interview
Semi-structured interviews	The patient participates in a one hour semi-structured interview. This takes place on the same day as the final objective measures. The questions explore the experience of any perceived cognitive issues as well as coping strategies and any other factors that may influence this experience. Within this interview there are is a short section asking questions regarding acceptability of the objective measures as part of the feasibility aspect of this study.	One hour

Qualitative Methods

The qualitative method of data collection will be a semi-structured interview which will take place at 28 days post start of treatment. The interview will be digitally audio recorded and transcribed verbatim. The purpose of the interview is to drill down into any issues identified by the quantitative assessments, but also to further elicit information regarding possible impact on the patient's life that this problem may have caused. The interviews are semi-structured but can be flexible so that they can be guided by any issues highlighted by the quantitative data collection. The interview schedule is in Appendix A.

The interview will be conducted by the primary researcher, in a private room and last for up to one hour. The participant will be told that they can have a break or stop the interview at any time. Interviewing patients on potentially sensitive issues can lead to an emotional response, either cathartic or distressing (38). At the end of the interview there will be two questions regarding the experience of the cognitive assessments; this will provide information regarding the tolerability of performing such structured assessments in this patient group.

Analysis plan

Research question one: Are targeted therapies given within the context of Phase I trials associated with deterioration in cognitive function.

The aim of these analyses are to describe patterns of decline and explore the use of methods in this patient group. In order to answer the first research question and provide a description of the function of the three cognitive domains from baseline across the timepoints. the following analysis plan will be conducted:

For peer review only - http://bmjopen.bmj.com/site/about/guidelines.xhtml

The HVLT-R, CTMT A and B and COWAT raw scores will be converted to age adjusted T scores using the professional manuals (39-42)

Sample means of the T scores at each timepoints will be presented with standard deviations and confidence intervals. Box plots will be produced to view the data. The proportions of patients showing CI (as defined in the methods of data collection section) will be reported as percentages.

To describe the change over time and comparison of means an ANOVA repeated measures test will be conducted. Whilst caution should be taken to interpret these results due to sample size the ANOVA may help to explore possible trends in cognitive function and provide effect sizes for the changes in the dependant variables (cognitive scores). The results from the ANOVA analyses will also be used to answer research question 3, more detail will be given about the independent variables to be included in the model.

Research question 2: What is the experience of cognitive function and the impact of any deterioration on the lives of these people on Phase I trials?

In order to answer the quantitative aspect of this question with the objective of assessing self-reported cognitive function the following analysis will be conducted. At each timepoint the global score will be calculated to according to the standard scoring protocol for FACT-cog (43). The proportions of patients reporting (PCI) on the PCI Subscale will be calculated at each time point using the cut-off point of >60 (44). Changes over time of perceived impairment will be evaluated using the established MCID of 9 points difference form baseline will be presented (45).

Research Question 3: What influence do potential confounding factors of depression, anxiety and fatigue have on the cognitive scores in this patient group?

To assess the association of potential confounding variables on cognitive decline in this patient group identified variables will be included in the ANOVA repeated measures. It is known there are some relationships between anxiety and depression, sex and fatigue with CI. It is also suggested in the literature that researchers investigating cognitive function should plan to account for these possible relationships. (46). Prior to inclusion in the ANOVA correlations between the DV and IV will be run to ensure that the model will be data driven rather than theory driven.

Bivariate correlation between baseline dependant variables and the independent variables will be conducted using the Pearson R standardised correlation. This will establish the variables required to be included in the ANOVA.

For peer review only - http://bmjopen.bmj.com/site/about/guidelines.xhtml

Calculating effect size

Effect size analysis using Cohen d will be conducted on all statistically significant changes from baseline to help understand the practical significance of the results. A small to moderate effect size is considered meaningful.

Research Question 3 and 4

Proportions of recruitment, refusal and attrition rates of the study will be presented as percentages. An 80% accrual rate is selected as a level to demonstrate acceptability to patients.

Proportions of correctly completely and interpreted nurse administered cognitive assessments will be recorded as agreed by clinical psychologist reported as overall number and percentages. It is expected that 90& agreement on cognitive scores to demonstrate accuracy.

Qualitative Data Analysis

The digital audio recording will be transcribed verbatim. The data will be organised using the Nvivo software package. The premise of thematic analysis is the examination and recognition of patterns within the data that are ultimately categorised into meaningful themes (42). Thematic analysis can use different approaches and be applied to varying types of data, it may be theory driven or content driven. In this instance it will be the content in the data that will drive the analysis (43).

Integration of data

At the stage of interpretation and reporting a mixed methods matrix will be utilised as a joint display of findings. This is a suggested practical way of viewing and analysing the different types of data together and offers a tool to guide the narrative during reporting (44,45). The comprehensive results and analysis will be held in a separate database. This matrix is to demonstrate findings in terms of any changes so that they can be considered during the interpretation stage in a holistic way taking into account both quantitative and qualitative findings.

Patient and Public Involvement

The protocol has had patient and public involvement in the design and development stage. The protocol framework was presented and discussed at a Royal Marsden Hospital Patient and Carer

For peer review only - http://bmjopen.bmj.com/site/about/guidelines.xhtml

For peer review only

Research Panel, PPI input ked to decisions regarding timings and numbers of assessments. A patient representative was consulted on and helped to develop the questions for the semi-structured interview.

Ethics and dissemination

The study has obtained Research Ethics Committee approval in the UK reference number: 18/LO.0084 and full Health Research Authority approval IRAS project number: 235668. The study is currently open to recruitment after a long pause in recruitment due to the COVID-19 pandemic.

Table and figures

Table 1 Research question aligned with objective, methods and analysis
Table 2 Methods of data collection

Figure 1 Patient pathway aligned with Phase I trial

References

1) Wong KM, Capasso A, Eckhardt SG. The changing landscape of phase I trials in oncology. Nat Rev Clin Oncol. 2016 Feb;13(2):106-17. doi: 10.1038/nrclinonc.2015.194. Epub 2015 Nov 10. PMID: 26552953.
2) Ivanova.A. (2007) Phase I trials in oncology *Wiley Encyclopedia of clinical trials* Wiley and sons.inc. Available at https://doi.org/10.1002/9780470181218.ch1
3) National Cancer Institute (2010) Common Terminology Criteria for Adverse Events (CTCAE) https://ctep.cancer.gov/protocoldevelopment/electronic_applications/ctc.htm
4) Ng. T. Cheung. Y. Ng. Q. Ho. H and Chan. A. (2014) Vascular Endothelial Growth Factor Inhibitors and Cognitive Impairment: Evidence and Controversies. *Expert Opinion on Drug Safety* 13 (1) DOI: 10.1517/14740338.2013.82803
5) Selamat. M. Loh. S. Mackenzie. L. Vardy. J (2014) Chemobrain Experienced by Breast Cancer Survivors: A Meta-Ethnography Study Investigating Research and Care Implications *PLoS ONE* 9(9) Available at: https://doi.org/10.1371/journal.pone.0108002
6) Fitch MI, Armstrong J, Tsang S. Patients' experiences with cognitive changes after chemotherapy. Can Oncol Nurs J. 2008 Fall;18(4):180-92. English, French. doi: 10.5737/1181912x184180185. PMID: 19580104.
7) Myers.C. and Perry.J. (2012) *Cognition and Cancer* Cambridge Medicine
8) Eysenck.M and Keane.M (2010) *Cognitive Psychology: A Student's Handbook.* Sixth Edition Psychology Press
9) Harvey PD. Domains of cognition and their assessment. Dialogues Clin Neurosci. 2019 Sep;21(3):227-237. doi: 10.31887/DCNS.2019.21.3/pharvey. PMID: 31749647; PMCID: PMC6829170.

For peer review only - http://bmjopen.bmj.com/site/about/guidelines.xhtml

10) Ahles TA. Brain vulnerability to chemotherapy toxicities. Psychooncology. 2012 Nov;21(11):1141-8. doi: 10.1002/pon.3196. Epub 2012 Oct 1. PMID: 23023994; PMCID: PMC3788849.
11) Boykoff N, Moieni M, Subramanian SK. Confronting chemobrain: an in-depth look at survivors' reports of impact on work, social networks, and health care response. J Cancer Surviv. 2009 Dec;3(4):223-32. doi: 10.1007/s11764-009-0098-x. Epub 2009 Sep 16. PMID: 19760150; PMCID: PMC2775113.
12) Martin M, Clare L, Altgassen AM, Cameron MH, Zehnder F. Cognition-based interventions for healthy older people and people with mild cognitive impairment. Cochrane Database Syst Rev. 2011 Jan 19;(1):CD006220. doi: 10.1002/14651858.CD006220.pub2. PMID: 21249675.
13) Gehring K, Roukema JA, Sitskoorn MM. Review of recent studies on interventions for cognitive deficits in patients with cancer. Expert Rev Anticancer Ther. 2012 Feb;12(2):255-69. doi: 10.1586/era.11.202. PMID: 22316373.
14) Ganz PA. "Doctor, will the treatment you are recommending cause chemobrain?". J Clin Oncol. 2012 Jan 20;30(3):229-31. doi: 10.1200/JCO.2011.39.4288. Epub 2011 Dec 19. PMID: 22184392.
15) Deeks ED. Olaparib: first global approval. Drugs. 2015 Feb;75(2):231-40. doi: 10.1007/s40265-015-0345-6. PMID: 25616434.
16) Moore. H and McSherry. W. (2013) Ethical Considerations in Translational Research. *Cancer Nursing* 12(10): 22-28

For peer review only

For peer review only - http://bmjopen.bmj.com/site/about/guidelines.xhtml

17) Wefel. J. Ahles. T. Vardy. J and Schagen. S (2011) International Cognition and Cancer Task Force recommendations to harmonise studies of cognitive function in patients with cancer *The Lancet Oncology* (12)7 703-708
18) Shilling V, Jenkins V, Morris R, Deutsch G, Bloomfield D. The effects of adjuvant chemotherapy on cognition in women with breast cancer--preliminary results of an observational longitudinal study. Breast. 2005 Apr;14(2):142-50. doi: 10.1016/j.breast.2004.10.004. PMID: 15767184.
19) Schagen SB, Muller MJ, Boogerd W, Mellenbergh GJ, van Dam FS. Change in cognitive function after chemotherapy: a prospective longitudinal study in breast cancer patients. J Natl Cancer Inst. 2006 Dec 6;98(23):1742-5. doi: 10.1093/jnci/djj470. PMID: 17148777.
20) Stewart A, Collins B, Mackenzie J, Tomiak E, Verma S, Bielajew C. The cognitive effects of adjuvant chemotherapy in early stage breast cancer: a prospective study. Psychooncology. 2008 Feb;17(2):122-30. doi: 10.1002/pon.1210. PMID: 17518411.
21) Ahles TA, Saykin AJ, McDonald BC, Li Y, Furstenberg CT, Hanscom BS, Mulrooney TJ, Schwartz GN, Kaufman PA. Longitudinal assessment of cognitive changes associated with adjuvant treatment for breast cancer: impact of age and cognitive reserve. J Clin Oncol. 2010 Oct 10;28(29):4434-40. doi: 10.1200/JCO.2009.27.0827. Epub 2010 Sep 13. PMID: 20837957; PMCID: PMC2988635.
22) Collins B, MacKenzie J, Tasca GA, Scherling C, Smith A. Cognitive effects of chemotherapy in breast cancer patients: a dose-response study. Psychooncology. 2013 Jul;22(7):1517-27. doi: 10.1002/pon.3163. Epub 2012 Aug 30. PMID: 22936651.
23) Collins B, Mackenzie J, Tasca GA, Scherling C, Smith A. Persistent cognitive changes in breast cancer patients 1 year following completion of chemotherapy. J Int Neuropsychol Soc. 2014 Apr;20(4):370-9. doi: 10.1017/S1355617713001215. Epub 2013 Nov 15. PMID: 24229809.
24) Jones D, Vichaya EG, Wang XS, Sailors MH, Cleeland CS, Wefel JS. Acute cognitive impairment in patients with multiple myeloma undergoing autologous hematopoietic stem cell transplant. Cancer. 2013 Dec 1;119(23):4188-95. doi: 10.1002/cncr.28323. Epub 2013 Sep 16. PMID: 24105672; PMCID: PMC3834212.
25) Cruzado J. Lopez- Santiago S. Martinez-Marin V. Jose-Moreno G. Custadio AB and Feliu J (2014) Longitudinal Study of cognitive dysfunctions induced by adjuvant chemotherapy in colon cancer patients. *Supportive Cancer Care* 22(7): 1815-1823
26) Kohli S, Griggs JJ, Roscoe JA, Jean-Pierre P, Bole C, Mustian KM, Hill R, Smith K, Gross H, Morrow GR. Self-reported cognitive impairment in patients with cancer. J Oncol Pract. 2007 Mar;3(2):54-9. doi: 10.1200/JOP.0722001. PMID: 20859374; PMCID: PMC2793748.
27) Quesnel C, Savard J, Ivers H. Cognitive impairments associated with breast cancer treatments: results from a longitudinal study. Breast Cancer Res Treat. 2009 Jul;116(1):113-23. doi: 10.1007/s10549-008-0114-2. Epub 2008 Jul 16. Erratum in: Breast Cancer Res Treat. 2009 Jul;116(1):129-30. PMID: 18629633.
28) Klaver KM, Duijts SFA, Engelhardt EG, Geusgens CAV, Aarts MJB, Ponds RWHM, van der Beek AJ, Schagen SB. Cancer-related cognitive problems at work: experiences of survivors and professionals. J Cancer Surviv. 2020 Apr;14(2):168-178. doi: 10.1007/s11764-019-00830-5. Epub 2019 Nov 25. PMID: 31768861; PMCID: PMC7182611.
29) Tricco AC, Lillie E, Zarin W, O'Brien KK, Colquhoun H, Levac D, Moher D, Peters MDJ, Horsley T, Weeks L, Hempel S, Akl EA, Chang C, McGowan J, Stewart L, Hartling L, Aldcroft A, Wilson MG, Garritty C, Lewin S, Godfrey CM, Macdonald MT, Langlois EV, Soares-Weiser K, Moriarty J, Clifford T, Tunçalp Ö, Straus SE. PRISMA Extension for Scoping Reviews (PRISMA-ScR): Checklist and Explanation. Ann Intern Med. 2018 Oct 2;169(7):467-473. doi: 10.7326/M18-0850. Epub 2018 Sep 4. PMID: 30178033.
30) Hlubocky FJ, Sachs GA, Larson ER, Nimeiri HS, Cella D, Wroblewski KE, Ratain MJ, Peppercorn JM, Daugherty CK. Do Patients with Advanced Cancer Have the Ability to Make Informed Decisions for Participation in Phase I Clinical Trials? J Clin Oncol. 2018 Aug

For peer review only

20;36(24):2483-2491. doi: 10.1200/JCO.2017.73.3592. Epub 2018 Jul 9. PMID: 29985748; PMCID: PMC6097830.

31) Jones RL, Serrano C, von Mehren M, George S, Heinrich MC, Kang YK, Schöffski P, Cassier PA, Mir O, Chawla SP, Eskens FALM, Rutkowski P, Tap WD, Zhou T, Roche M, Bauer S. Avapritinib in unresectable or metastatic PDGFRA D842V-mutant gastrointestinal stromal tumours: Long-term efficacy and safety data from the NAVIGATOR phase I trial. Eur J Cancer. 2021 Mar;145:132-142. doi: 10.1016/j.ejca.2020.12.008. Epub 2021 Jan 16. PMID: 33465704.
32) McKay RR, De Velasco G, Werner L, Bellmunt J, Harshman L, Sweeney C, Rosenberg JE, Hirsch M, Signoretti S, Van Allen EM, Walsh M, Vaishampayan U, McDermott DF, Choueiri TK. A phase 1 study of buparlisib and bevacizumab in patients with metastatic renal cell carcinoma progressing on vascular endothelial growth factor-targeted therapies. Cancer. 2016 Aug 1;122(15):2389-98. doi: 10.1002/cncr.30056. Epub 2016 May 19. PMID: 27198170; PMCID: PMC5567751.
33) Wicki A, Brown N, Xyrafas A, Bize V, Hawle H, Berardi S, Cmiljanović N, Cmiljanović V, Stumm M, Dimitrijević S, Herrmann R, Prêtre V, Ritschard R, Tzankov A, Hess V, Childs A, Hierro C, Rodon J, Hess D, Joerger M, von Moos R, Sessa C, Kristeleit R. First-in human, phase 1, dose-escalation pharmacokinetic and pharmacodynamic study of the oral dual PI3K and mTORC1/2 inhibitor PQR309 in patients with advanced solid tumors (SAKK 67/13). Eur J Cancer. 2018 Jun;96:6-16. doi: 10.1016/j.ejca.2018.03.012. Epub 2018 Apr 13. PMID: 29660598.
34) Basch E, Jia X, Heller G, Barz A, Sit L, Fruscione M, Appawu M, Iasonos A, Atkinson T, Goldfarb S, Culkin A, Kris MG, Schrag D. Adverse symptom event reporting by patients vs clinicians: relationships with clinical outcomes. J Natl Cancer Inst. 2009 Dec 2;101(23):1624-32. doi: 10.1093/jnci/djp386. Epub 2009 Nov 17. PMID: 19920223; PMCID: PMC2786917.
35) Veitch ZW, Shepshelovich D, Gallagher C, Wang L, Abdul Razak AR, Spreafico A, Bedard PL, Siu LL, Minasian L, Hansen AR. Underreporting of Symptomatic Adverse Events in Phase I Clinical Trials. J Natl Cancer Inst. 2021 Aug 2;113(8):980-988. doi: 10.1093/jnci/djab015. PMID: 33616650; PMCID: PMC8502480.
36) Sailors MH, Bodurka DC, Gning I, Ramondetta LM, Williams LA, Mendoza TR, Agarwal S, Sun CC, Cleeland CS. Validating the M. D. Anderson Symptom Inventory (MDASI) for use in patients with ovarian cancer. Gynecol Oncol. 2013 Aug;130(2):323-8. doi: 10.1016/j.ygyno.2013.05.009. Epub 2013 May 15. PMID: 23685012; PMCID: PMC3713195.
37) Upadhayay N, Guragain S. Comparison of cognitive functions between male and female medical students: a pilot study. J Clin Diagn Res. 2014 Jun;8(6):BC12-5. doi: 10.7860/JCDR/2014/7490.4449. Epub 2014 Jun 20. PMID: 25120970; PMCID: PMC4129348.
38) Ryan, Francis; Coughlan, Michael & Cronin, Patricia (2009). *Interviewing in qualitative research: The one-to-one interview.* International Journal of Therapy and Rehabilitation, *16*(6), 309
39) Brandt. J. and Benedict.R. (2001) Hopkins Verbal Learning Test-Revised- Professional Manual Psychological Assessment Resources
40) Benton A, Hamsher Brandt. J. and Benedict.R. (2001) Hopkins Verbal Learning Test-Revised-Professional Manual Psychological Assessment Resources
41) Benton, Hamsher and Sivan. A (1978) Multilingual Aphasia Examination Manual of Instructions 3rd Edition Psychological Assessment Resources
42) Reynolds C (2002) Comprehensive Trail-Making Test Examiners Manual Pro-Ed
43) Cella DF, Tulsky DS, Gray G, Sarafian B, Linn E, Bonomi A, Silberman M, Yellen SB, Winicour P, Brannon J, et al. The Functional Assessment of Cancer Therapy scale: development and validation of the general measure. J Clin Oncol. 1993 Mar;11(3):570-9. doi: 10.1200/JCO.1993.11.3.570. PMID: 8445433.

For peer review only - http://bmjopen.bmj.com/site/about/guidelines.xhtml

44) Dyk KV, Crespi CM, Petersen L, Ganz PA. Identifying Cancer-Related Cognitive Impairment Using the FACT-Cog Perceived Cognitive Impairment. JNCI Cancer Spectr. 2019 Nov 29;4(1):pkz099. doi: 10.1093/jncics/pkz099. PMID: 32064458; PMCID: PMC7015054.

45) Cheung YT, Foo YL, Shwe M, Tan YP, Fan G, Yong WS, Madhukumar P, Ooi WS, Chay WY, Dent RA, Ang SF, Lo SK, Yap YS, Ng R, Chan A. (2014) *Minimal clinically important difference (MCID) for the functional assessment of cancer therapy: cognitive function (FACT-Cog) in breast cancer patients.* J Clinical Epidemiology 67(7):811-20. doi: 10.1016/j.jclinepi.2013.12.011. Epub 2014 Mar 18. PMID: 24656406.

46) Lange, F. Joly, J Vardy, G. Winocur, M.B. De Ruiter, H. Castel *(2019) Cancer-related cognitive impairment: an update on state of the art, detection, and management strategies in cancer survivors* Annals of Oncology *(30) 12 P1925-1940 DOI: https://doi.org/10.1093/annonc/mdz410*

47) Guest. G. (2012) *Applied Thematic Analysis* Thousand Oaks California

48) Braun V and Clarke V (2006) Using thematic analysis in psychology. *Qualitative Research in Psychology* 3(2): 77-101

49) Johnson R. Onwuegbuzie A and Turner. L. (2007) Toward a definition of mixed methods research. *Journal of mixed methods* 1(2): 112-133

50) Onwuegbuzie AJ, Slate JR, Leech NL, Collin. N Kathleen. M (2009) *Mixed data analysis: Advanced integration techniques.* International Journal of Multiple Research Approaches 3(1): 13–33.

Author's Contributions

Sarah Stapleton – Primary Researcher, Research design concept, Protocol Development and Writing

Prof. Anne-Sophie Darlington- PhD primary supervisor, Protocol Development

Prof. Johann De-Bono – Clinical oversight of the study as head of unit, review of protocol development

Prof. Theresa Wiseman – Principal Investigator , PhD supervisor, Protocol development ,

Competing Interests

Sarah Stapleton None declared however SS post is funded by Cancer Research UK

Prof. Anne-Sophie Darlington None declared

Prof. Theresa Wiseman None declared

Prof. Johann De-Bono: JDB has served on advisory boards and received fees from many companies including Astra Zeneca, Astellas, Bayer, Bioxcel Therapeutics, Boehringer Ingelheim, Cellcentric, Daiichi, Eisai, Genentech/Roche, Genmab, GSK, Janssen, Merck Serono, Merck Sharp & Dohme, Menarini/Silicon Biosystems, Orion, Pfizer, Qiagen, Sanofi Aventis, Sierra Oncology, Taiho, Vertex Pharmaceuticals. He is an employee of The ICR, which have received funding or other support for his research work from AZ, Astellas, Bayer, Cellcentric, Daiichi, Genentech, Genmab, GSK, Janssen, Merck Serono, MSD, Menarini/Silicon Biosystems, Orion, Sanofi Aventis, Sierra Oncology, Taiho, , Pfizer, Vertex, and which has a commercial interest in abiraterone, PARP inhibition in DNA repair defective cancers and PI3K/AKT pathway inhibitors (no personal income). He was named as an inventor, with no financial interest, for

For peer review only - http://bmjopen.bmj.com/site/about/guidelines.xhtml

patent 8,822,438. JDB is a National Institute for Health Research (NIHR) Senior Investigator. The views expressed in this article are those of the author(s) and not necessarily those of the NHS, the NIHR, or the Department of Health.

Funding Statement

The research received no specific grant from any funding agency in the public, commercial or not for profit sectors. However the primary researcher's post is funded by Cancer Research UK.

Acknowledgements

The author would like to acknowledge the Royal Marsden Patient and Carer Research Review Panel for their contribution to the protocol development.

-

For peer review only

Figure 1 Patient pathway on study aligned with Phase I trial

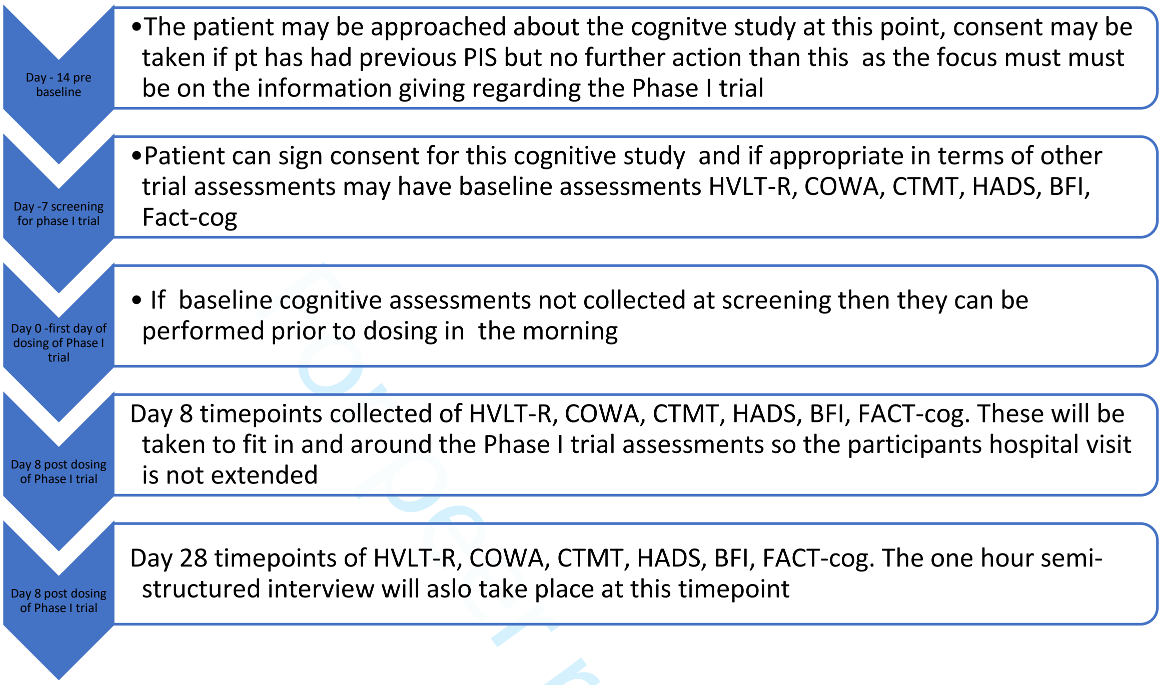

For peer review only

Appendix A Interview Schedule

Interviewer to introduce themselves to the participant, clarify that they know why they are here and that consent still stands. Give examples of phrasing of questions and suggested idea of how long the interview will last. Remind the participant that they can stop the process if they wish. Recheck that they are happy for the audio recording to start, and they are ready to begin the interview.

Using open ended questions, the Interview will cover the following at least but not exclusively the following areas:

Memory

Concentration

Ability to speak/articulate/communicate

General Health

Impact of trial treatment/schedule

Mood

The first two questions will be general to help the patient to start talking and feel relaxed in the environment

1) Tell me how you have been since you started the trial

2) Can you tell me of any changes you have noticed since starting the trial?

If the participant mentions any cognitive or related symptoms, they will be asked to expand on this, using such phrases such as

- "Can you tell me a bit more about that?"
- "What was that like for you?"
- "How did you deal with that? "

If not questions regarding domains of cognitive function will be asked – examples of this include:

"Tell me about how your memory has been since you started the trial"

"tell me how your concentration has been since starting on the trial? "

For peer review only

"Have you noticed any differences in speaking or finding the right words sometimes?"

"Have you noticed any changes in your ability to think straight or work things out?"

"Have you had any difficulty relating to this? "

"How has your mood been since starting on the trial drug?"

If any changes or symptoms are discussed ask patient to elaborate – when did it start, how does it manifest/feel/ present? – Characterise the symptom with the participant onset, severity, duration, improvements, deterioration etc. Examples of questions to elaborate on symptoms are:

"How did you first notice this?"

"Tell me a bit about what happened to you "

"How did you feel when this was happening?"

Other questions will then cover the areas that are relevant to the investigation of this study. If patients have discussed specific symptoms the following questions will be asked in relation to this.

"Tell me how this has impacted on your life?"

"Has this affected you at work/home/ your relationships with others in any way?"

Once the impact and effect on certain areas has been established try to prompt further to explore how this made the patient feel and how they cope with this.

"Did you try anything to help the situation? "

"Was this a difficult thing to cope with?"

Dependant on what the interviewee discusses during the interview, complete with an exploration of strategies or useful interventions that have or may have helped covering:

"Would they have been anything useful to know about pre- treatment to help you prepare for any of these symptoms?"

"Tell me about any strategies that you have used yourself to help you overcome these problems?"

"tell me of anything the team here could have done to help you manage the ……. You discussed before "

For peer review only

The last two questions are regarding the acceptability of the assessments performed for this study- these questions will be asked to all participants.

"How have you found the extra cognitive assessments that have been performed for this study?"

"Would you be happy to have these assessments performed alongside treatment as the norm?"

End the interview by checking if there is anything that the participant wants to add. Ask if they are happy for the interview to be used. Thank the participant for taking part.

For peer review only

BMJ Open

What is the impact of targeted therapies given within Phase I trials on the cognitive function of patients with advanced cancer. A mixed methods exploratory study conducted in an early clinical trials unit.

Journal:	*BMJ Open*
Manuscript ID	bmjopen-2021-050590.R2
Article Type:	Protocol
Date Submitted by the Author:	21-Jun-2022
Complete List of Authors:	Stapleton, Sarah ; Royal Marsden Hospital Sutton, Drug Development Unit ; University of Southampton, Faculty of Health Sciences<br>Darlington, Anne-Sophie; University of Southamptom, Faculty of Health Sciences<br>de Bono, J. S.; Royal Marsden NHS Fdn Trust; Institute of Cancer Research Division of Cancer Therapeutics<br>Wiseman, Theresa; The Royal Marsden NHS Foundation Trust, ; University of Southampton, Faculty of Health Sciences
**Primary Subject Heading**:	Oncology
Secondary Subject Heading:	Pharmacology and therapeutics
Keywords:	CLINICAL PHARMACOLOGY, Adult oncology < ONCOLOGY, Delirium & cognitive disorders < PSYCHIATRY, Clinical trials < THERAPEUTICS

SCHOLARONE™
Manuscripts

Cognitive Function Protocol for submission to BMJ

Title: What is the impact of targeted therapies given within Phase I trials on the cognitive function of patients with advanced cancer. A mixed methods exploratory study conducted in an early clinical trials unit.

Short Title: Cognitive Function and Phase I cancer drug trials

Authors:
Sarah. E. Stapleton (1) Prof. Anne-Sophie Darlington (2) Prof. Johann De-Bono (1,3) Prof.Theresa Wiseman (1)

1 Royal Marsden Hospital Downs Rd Sutton Surrey Sm2 5PT

2 University of Southampton University Rd Southampton SO17 1BJ

3 Institute of Cancer Research 15 Cotswold Rd Sutton SM2 5NG

Corresponding Author: Sarah Stapleton Royal Marsden Hospital Downs Rd Sutton Surrey SM2 5PT sarah.stapleton@rmh.nhs.uk

Keywords: Drug Development , Early Phase I Clinical Trials, Cognitive function, Cognitive impairment, Cognitive assessment

For peer review only

Cognitive Function Protocol for submission to BMJ

Abstract

Introduction: Novel therapies such as small protein molecule inhibitors and immunotherapies are tested in early phase trials before moving to later phase trials and ultimately standard practice. A key aim of these clinical trials is to define a toxicity profile, however the emphasis is often on safety with measurements of organ toxicity. Other subjective side effects can be underreported because they are not measured formally within the trial protocols. The concern from clinical practice is that cognitive toxicity is poorly studied and may be under-reported in this context. This could lead to toxicity profiles of new treatments not being fully described and patients with unmet need in terms of acknowledgement and support of symptoms. This protocol outlines a framework of an exploratory study with feasibility aspects to investigate the impact and experience of cognitive changes for patients on Phase I trials.

Methods and analysis: This is a mixed methods study combining quantitative and qualitative approaches. The sample is thirty Patients with advanced cancer who are participating in Phase I trials of novel therapies in the early clinical trials unit of a specialist cancer centre. A test battery of validated cognitive assessments will be taken alongside patient reported outcome measures at three time-points from baseline, day eight and day 28 post start of treatment. At day 28 a semi structured interview will be conducted and the narrative thematically analysed. Results will be integrated to offer a comprehensive description of cognitive function in this patient group.

Ethics and dissemination: The study has received full HRA and ethical approval. It is the first study to introduce formal cognitive assessments in a cancer Phase I trial context. The study has the potential to highlight previously unreported side effects and more importantly unmet need in terms of care for our patients who are participating in the trials.

For peer review only

Strengths and Limitations

- This is the first study to investigate cognitive function longitudinally during the course of Phase I trials.
- The findings of the trial will establish feasibility of conducting formal and consistent cognitive testing in early clinical drug trial protocols.
- Describing the symptoms of patients in this group will help to identify unmet need in terms of support and management.
- Recommendations of the International Cancer and Cognition Task Force to harmonise research in this field were adopted in the trial design.
- Due to the exploratory nature and feasibility aspects of the study, the sample size is not statistically powered. A future study would benefit from a powered calculated sample size to demonstrate any treatment related change with more certainty.

Cognitive Function Protocol for submission to BMJ

Introduction

In cancer drug development advances in the understanding of genetics and the biochemical pathways involved in cancer have helped researchers to rationally design “molecularly targeted drugs”. These novel targeted therapies and immunotherapies are first tested clinically in patients within phase I trials in cancer centres. Due to the experimental nature of phase I trials and the fact that little is known about the toxicities, patients need to have exhausted all standard treatment options to participate. To contextualise, these patients will generally have advanced cancer and pre-treated disease. It is a heterogenous patient population in terms of tumour types. However, what the patients do have in common is a shared experience of the continuous cycle of treatment and disease progression to the point where there is no further treatment available. Patients on phase I trials often have significant tumour burden with metastatic disease and a life limiting prognosis. These patients remain motivated to participate in phase I trials of experimental therapies and despite having advanced progressive disease the criteria for participating in phase I means the patients do have to have a certain level of fitness to meet eligibility requirements of specific protocols.

At this stage of drug development, the main aims are to find the optimal dose, investigate the pharmacology and to define the toxicity profiles (1). When considering toxicity profiles of these potential new treatments the emphasis is on safety and this is reflected in many of the standard assessments in the protocols such as cardiology monitoring and renal and liver function tests (2). Clinicians and nurses involved in the conduct of phase I trials do perform medical history taking and nursing assessments to gain a full perspective of other side effects reported but they are interpreted and recorded in a subjective manner usually using the Common Terminology Criteria for Adverse Events (CTCAE) which is a National Institutes of Health – National Cancer Institute, Division of Cancer Treatment and Diagnosis, Cancer Therapy Evaluation Program initiative. It is a multi-dimensional tool designed specifically for use in clinical trials. The tool enables the clinician to categorise symptoms that are graded 0-5, translating respectively to none, mild, moderate, severe, life threatening and death. The use of the CTCAE is commonplace in all phase I trials (3).

This exploratory study investigates the impact of targeted therapies within the context of an experimental trial on the cognitive function of patients with advanced cancer. It is suspected that some of the targeted therapies tested within phase I trials may have a negative impact on the patient’s cognitive function. Evidence from clinical practice suggests that patients are experiencing symptoms relating to cognitive function, however this does not seem to translate to reports of cognitive toxicity

For peer review only - http://bmjopen.bmj.com/site/about/guidelines.xhtml

within clinical trial data. As cognitive function is not formally assessed the extent, nature or domain of cognitive impairment is not well described. There is also suggestion in literature that the difficulty in ascertaining the relationship between cognitive impairment and targeted therapies may be due to the underreporting of cognitive symptoms in clinical trials (4,5).

Rationale

Impairment of cognitive function in the field of traditional cytotoxic chemotherapy has been investigated and it is known that such a symptom impairs quality of life (6). Therefore, if cognitive function was investigated and described in the context of patients on phase I trials, it could provide a basis of knowledge on which to develop interventions and coping strategies with the aim of preventing or ameliorating the effects of this possible decline in cognitive function.

Cognitive function is described as the internal processes involved in making sense of the world and the capacity to make decisions (7). Such processes include attention, perception, learning, memory, language, problem solving, reasoning, and thinking (8). These functions are defined in terms of domains that can broadly be divided into sensory and motor skills, memory, attention and concentration, executive function (which includes reasoning and problem solving) and language and verbal skills (9). Whilst the domains overlap in terms of function each can be assessed using validated cognitive assessment tools. Impaired cognitive function in cancer patients can present as loss of memory, difficulty with word finding, loss of concentration and difficulty in task switching (10). Even though cognitive function is not assessed as part of the phase I trials, anecdotal reports from some patients suggest that they experience some deterioration whilst on the trial which is often temporal to dosing of drug.

For patients this has implications in that they may have a symptom they feel unable to discuss. This in turn can mean that they do not receive any help or support regarding the problem which can be isolating and frightening for the patient (11). Not having the symptom acknowledged or having no information to warn about the possibility of it happening has been reported as being distressing and difficult for patients. (11,12).

Implications on a broader scale will mean that better defined toxicity profiles will improve understanding of consequences of treatments. This will assist in developing interventions that help patients make informed choices about having certain treatments and be better equipped to cope with

For peer review only - http://bmjopen.bmj.com/site/about/guidelines.xhtml

the consequences of them. This may be as simple as recognition of the potential problem with information and preparation for the patient or the implementation of strategies to help with symptoms such as compensatory memory techniques, rehabilitation and relaxation (13,14).

In terms of relevance the numbers of patients initially receiving such treatments in phase I trials may be relatively small, usually tens to hundreds. Nevertheless, for these patients with a limited prognosis issues regarding side effects are important. Once a drug has gone through the phases of testing and is licensed for practice the numbers of patients receiving these treatments could then become thousands (15). The number of cancer patients participating in clinical trials is increasing (16). Therefore, it is important to find out accurately what is happening to patients on clinical trials so they can be supported with the management of side effects such as diminished cognitive function. In order to begin to address this and improve the care and understanding of patients on phase I trials this proposed study will be the first exploration using formal assessment of cognitive function in this context.

Evidence Gap

Early work in chemotherapy related cognitive impairment highlighted a disparity in trial designs and some limitations in research in this field such as the use of cross-sectional trial design offering no baseline from which to measure change. Lack of consistency in the type of cognitive assessment methods used in the trials also lead to broad differences in reporting of the prevalence of cognitive impairment (CI). This led to a team of expert cognitive researchers founding a multidisciplinary group known as the International Cancer and Cognition Task Force (ICCTF). The ICCTF was founded in 2004 and one of the aims was to produce guidelines to harmonise study design. The recommendations were to include pre-treatment assessment, longitudinal follow up, the use of a control group and a core set of cognitive assessment tests when investigating cognitive function in cancer patients (17). All recommendations will be adopted for this protocol except the use of a control group. It would be difficult to match a control for this patient group as the main reason that patients do not enter a Phase I trial is due to a deterioration in clinical condition. Therefore, patients in a comparable situation would likely be receiving palliative care only and it may not be appropriate to ask them to participate as a control.

The subsequent studies that followed these guidelines continued to show associations between systemic chemotherapy treatment and CI as well as discovering that patients with cancer often have a degree of CI at baseline when compared to matched healthy controls (18 -20). The studies also

For peer review only - http://bmjopen.bmj.com/site/about/guidelines.xhtml

describe the manifestation of CI as a decline in concentration, memory, verbal fluency and executive functioning which includes higher order cognitive abilities of planning and abstract thinking. Due to the longitudinal design some have been able to demonstrate compelling evidence of dose dependent relationships between treatment and severity of diminished cognitive function (21-25).

Some of the studies also highlighted the value of collecting the patient's own perception of cognitive function as they showed that this can vary from the validated cognitive assessment measures, (26,27). What is not addressed in many of these studies is the meaning to the patient in terms of how it may affect their lives. Just a small number of qualitative studies have been conducted exploring the impact of cognitive function on the quality of life of patients. They show that there is a negative effect on work, being unable to return to previous occupations and detrimental effects on emotional, social and psychological aspects of daily life (28)

A scoping literature review was conducted with the aim of understanding the status of assessment and reports of cognitive function within phase I trials. The scoping review utilized the Preferred Reporting Items for Systematic reviews and Meta-Analyses extension for Scoping Reviews (PRISMA-ScR) checklist as a framework (29). Papers were included if they reported cognitive symptoms or symptoms categorised as cognitive within the toxicity reports of the trial. The purpose of this was to identify any issues relating to cognitive function including how it was assessed and reported. Also included were any studies investigating cognitive function in a phase I population. Other observational studies that explored symptom presentation and reporting in phase I trials were included. The purpose of this was to understand any factors that are specific to this patient population and context that may influence identification of cognitive symptoms. Excluded were studies that did not include cognitive issues in the reports. Studies of patients with central nervous system tumours and paediatric patients were excluded as due to the nature of the disease and treatment there are cognitive issues specific to each of these patient's groups. Search limits included papers in English and dates of publication 2010-2021.

Just one study in the review specifically investigated cognitive function in a population of phase I patients. The aim was to identify the relationship between cognitive function and intact decisional capacity in relation to informed consent. The cross-sectional design assessed the cognitive function of patients at baseline prior to starting the trial so there was no longitudinal assessment or feasibility assessment throughout the course of the clinical trial. The sample was representative of a phase I population, outcome measures were valid, and associations of confounding variable were adjusted

For peer review only - http://bmjopen.bmj.com/site/about/guidelines.xhtml

for in the analyses. The research team found that a proportion of patients had cognitive impairment at baseline and those with worse cognitive function were less likely to understand and remember some of the key principles of the trial. The study was limited due to the cross-sectional design and the researchers do propose that further longitudinal work assessing cognitive function in patients on Phase I trials would be of value (30).

Evidence of cognitive toxicity reports in the phase I trials was limited. A small number did report cognitive toxicity. The symptoms were not well characterised and sometimes aggregated or reported as a cognitive category rather than the symptom, making it difficult to understand exactly what the adverse events were.

A phase 1 trial investigating safety and efficacy of BLU-285, a highly selective targeting inhibitor of mutant KIT and PDGFR in a population of 116 patients with Gastro-Intestinal Stromal Tumour reported cognitive impairment as a toxicity. The symptoms are aggregated in the report as cognitive effects. The paper reports that 46% (n=115) have all grade cognitive effects and group together memory impairment, confusional state, cognitive disorder and encephalopathy within this category (31). This perhaps represents a lack of clarity in working definitions of cognitive impairments.

Other reports of cognitive toxicity within phase I include a combination study investigating the maximum tolerated dose and toxicity profile of VEGF inhibitor and Pi3K inhibitor for patients did not include cognitive assessments but did include specific mental health diagnostic tools in the trial design. Despite the lack of cognitive assessments 10 %(n=30) of patients on the trial reported cognitive disturbance. This is another example of the symptom being reported as a category rather than the actual symptom, hence the presentation of the cognitive disturbance is impossible to elucidate. This vague description leads to a poor understanding of the symptom which is attributed as a severe side effect of treatments. Indeed the cognitive disturbance was graded severe enough to be classified as a dose limiting toxicity. A dose limiting toxicity is a drug related adverse event pre-defined in the protocol and is significant as it may prevent further dose escalation. This means that the recommended doses could be affected (32).

Despite not including any cognitive assessments within the trial protocol, a phase I trial of a Pi3K and mTor inhibitor in combination also reported" cognitive disorders " in some patients on the trial this is further categorised as memory loss, confusional state, attention deficit and agitation. Causality was attributed to the investigational medicinal product (33). Because no formal cognitive assessments were incorporated into the study design, the severity and duration of this is unclear.

For peer review only - http://bmjopen.bmj.com/site/about/guidelines.xhtml

None of the papers filtered in the review used formal cognitive assessments within the trial protocol, so the review confirms that there are no formal or consistent cognitive assessments as part of current standard practice within early clinical trials.

More recently studies are emerging that look at Patient Reported Outcome Measures (PROMs) in phase I trials, in particular the PROMs version of the CTCAE (PRO-CTCAE) which is a multidimensional PROMs specifically designed by the NCI to capture symptomatic adverse events in clinical trials (34). Whilst its use is commonplace in phase 2 and 3 trials it's used less in early phase trials where definition of toxicity is a key endpoint (35). One study recruited 265 patients on phase I trials and administered the PROMs at three timepoints. In the case of cognitive events there are two items on the scale of memory impairment and impaired concentration. The study team describe a difference between PROMs and clinician reports as 25.9% of patient reports of memory as compared to 0.5% of clinicians and in the case of impaired concentration 0.7 to 28.1 % respectively. A similar study using the MD Anderson symptom inventory (MDASI) which is also a patient reported questionnaire focusing on 13 core common cancer symptoms (36). It has one item relating to cognitive function labelled as "difficulty remembering". This study showed that up to 41% of patients identified as having difficulty remembering.

The number of experiences of cognitive impairment in the PROMs studies are incongruous with the many papers filtered in this review where there are simply no or very limited reports of CI. This highlights potential unmet need in this patient group.

In summary, there is a lack of evidence to show the effects on cognitive function for patients on phase I clinical trials. This has a two-fold consequence. Firstly, that the side effects of the drugs may not be well characterized and only become apparent in latter stages of drug development or even when a drug becomes standard practice. Secondly patients who are participating in phase I trials may have unmet need in terms of identification and support of symptoms of CI. This research protocol outlines an exploratory framework for a study utilizing mixed methods to both identify the existence of cognitive decline in these patients but also describe the impact this has on their everyday lives.

For peer review only - http://bmjopen.bmj.com/site/about/guidelines.xhtml

3. Research Question and Aims

The study is not hypothesis testing and is framed as an exploratory study. The overarching aim is to identify, characterise and gain an understanding of the effects on the cognitive function of patients on novel targeted therapies given within the context of phase I trial. The study has an added feasibility aspect to gain an understanding of how acceptable and appropriate nurse led cognitive assessments are in this patient group bearing in mind the existing potential burden of the phase I trial assessments. The overarching research question for the study is:

"What is the impact of the targeted therapies given within the context of a Phase I trial on the cognitive function of patients with advanced cancer?"

The research question can be broken down into six sub questions. The research sub questions are presented below aligned with the objectives, methods and analysis (Table 1).

For peer review only

Table 1: Research questions aligned with objectives, methods and analyses.

**Research Question (RQ))**	**Objectives**	**Methods of Data Collection**	**Analysis**
RQ1: What is the cognitive function of the patients on Phase I trials of novel therapies	To describe the cognitive function over time of this patient group	Cognitive assessments of memory (Hopkins Verbal Learning Test-r) executive function (Comprehensive Trail Making Test A and B) and verbal fluency (Controlled Oral Word Association Test)	Age adjusted T scores will be presented as means and standard deviations with confidence intervals for each timepoint. Proportions of patients with CI will be presented at each timepoint.<br><br>ANOVA repeated measures will be conducted to describe the change over the three timepoints.
RQ2: What is the experience of cognitive function and the impact of any deterioration on the lives of these people on Phase I trials?	To explore and understand the patient narrative surrounding cognitive function in this context.	Individual in-depth interviews<br><br>PROMs of perceived CI Fact-cog	Thematic analysis (Braun and Clark)<br><br>Decline at each timepoint will be present as predefined Minimum Clinically Important Differences (Cut off points are available for perceived impairment subscale that can be used to present proportions of patients with impairment at each timepoint).
RQ3: Do patient's perspectives mirror cognitive outcome measures overtime?	To compare, contrast and look for confirmation, divergence and an in-depth characterization of cognitive function in this patient group	Combined qual and quant	Integration at analysis stage using mixed methods matrix to interpret findings
RQ4 What influence do potential confounding factors of depression, anxiety and fatigue have on the cognitive scores in this patient group?	To assess the impact of potential confounding variables on cognitive decline in this patient group.	PROMs of fatigue, anxiety and depression	ANOVA will be conducted to examine the influence of the confounding variables
RQ5: How acceptable are the cognitive assessments alongside the phase I clinical trial?	To measure acceptability of extra cognitive assessments alongside Phase I trials.	Recruitment, refusal and attrition rates recorded<br><br>Qualitative enquiry as part of the interview to ask how acceptable the cognitive assessment battery has been	Measurements of uptake of trial, refusal to enter study (with reasons if possible) attrition rates (presented as numbers and percentages)<br>Frequency of positive responses in qualitative questions<br>Coding and thematic analysis of narrative response to enquiry about acceptability of questions.
RQ6: How accurate are nurse led cognitive assessments (the nurse has accredited training in Psychometric testing and is being supervised by a clinical psychologist).		Measurement of number of approved accurate cognitive assessments undertaken as validated by psychologist.	Percentages of positive validation by supervising psychologist.

For peer review only - http://bmjopen.bmj.com/site/about/guidelines.xhtml

Methods and Analysis

This is a mixed methods longitudinal study design using both qualitative and quantitative approaches with equal weighting.

Sample

A non-probability sampling strategy technique of convenience sampling will be used. Most of the patients entering a phase I trial will be eligible for this study therefore this sampling technique will allow for the quickest access to patient recruitment. The patients will be recruited from the clinical trials unit within a specialist cancer hospital, where they attend on a weekly basis. The patient group is heterogeneous in terms of tumour type, age and gender. However, the similarities within the sample population will be the stage of disease, the types of previous treatment and context. Although a convenience sample will be used there will be some stratification in terms of gender. Due to sample size any stratification of other possible confounding factors such as age, educational status will not be possible. Age and educational status (as a key component of cognitive reserve) have been shown to have an influence of altered cognitive function. The sample split between male and female will aim to be equal as it is known that there are cognitive differences relating to gender (37). As this is an exploratory study with feasibility aspects, the sample size will be 30 patients.

Inclusion Criteria

- Patients with a confirmed diagnosis of cancer
- Patients who are eligible for the trial and meet the trial specific eligibility criteria
- Patients who are allocated to and commence a Phase I trial (either IV or oral)
- Patients who are over eighteen

Exclusion Criteria

- Patients with a primary central nervous system tumour
- Patients who have received whole brain radiotherapy
- Patients who are non-English speaking
- Patients with uncontrolled psychiatric disorders

Patient pathway

The following figure 1 describes the patient pathway. This is their schedule of assessments aligned with the Phase I clinical trial pathway and schedule of assessments.

Insert Figure 1 Patient pathway aligned with Phase I trial

For peer review only - http://bmjopen.bmj.com/site/about/guidelines.xhtml

Quantitative Methods

This study follows the ICCTF recommendations of a core battery of validated cognitive assessments of memory, verbal fluency and executive functioning at baseline, day eight and day 28 post start of trial treatment. As well as the cognitive assessments that will be conducted by the primary researcher there will be PROMs of fatigue, depression and anxiety and self- perception of cognitive function. The methods of data collection and administration are summarized below (Table 2).

Table 2: Methods of data collection and administration

**Validated Cognitive Assessment Tests**	**Process**	**Time taken to complete test**
Controlled Oral Word Association **COWA** (35)	This is an oral fluency test. The assessment is made by asking the participant to make verbal associations to letters of the alphabet by saying as many words as possible one can think beginning with a given letter. A sheet is available to record verbatim response. Total number of acceptable responses provides the raw score.	5-10 minutes
Hopkins Verbal Learning Test _Revised **HVLT_R (36)**	This is a word list learning and memory test. Each form of the test consists of 12 nouns with 4 items taken from each semantic category. The word list is read to the patient who then must recall as many words as possible. The task is repeated two or three times. A 25-minute interval is given after which a delayed recall test is performed. The tests are then scored in terms of total recall, delayed recall and retention %.	40 mins (including 25 minutes for delayed recall test)
Comprehensive Trail Making Test **CTMT (37)**	This test measures executive function, concentration and task switching. The test consists of a standardised set of visual search and sequencing tests. The raw score for each trail test is the number of seconds required for the participant to complete; time is added per error as the examiner corrects them.	5-12 minutes
**Patient Reported Outcomes**	**Process**	**Time taken to complete test**
**Fact-Cog** (38)	This is a 35-item questionnaire measuring the patient's own perception of cognitive function changes. It is based on a 5-point rating scale "in the last 7 days timeframe".	15 minutes
Hospital Anxiety and Depression scale **HADS (39)**	The hospital anxiety and depression score is a 14 item scale for patients who may have other medical problems. It is essentially a screening tool for anxiety and depression. 7 items cover anxiety, and 7 items cover depression. Each item has score of 0-3 making a potential score between 0-21 for each problem.	15 mins
Brief Fatigue Inventory **BFI (40)**	The brief fatigue inventory is a six-item assessment tool measuring the patients' levels of fatigue. It records changes over short periods with a focus on fatigue in the previous 24 hours.	5 mins
**Qualitative data collection**	**Process**	**Time taken to complete interview**
Semi-structured interviews	The patient participates in a one-hour semi-structured interview. This takes place on the same day as the final objective measures. The questions explore the experience of any perceived cognitive issues as well as coping strategies and any other factors that may influence this experience. Within this interview there is a short section asking questions regarding acceptability of the objective measures as part of the feasibility aspect of this study.	One hour

For peer review only - http://bmjopen.bmj.com/site/about/guidelines.xhtml

Qualitative Methods

The qualitative method of data collection will be a semi-structured interview which will take place on day 28. The interview will be digitally audio recorded and transcribed verbatim. The purpose of the interview is to drill down into issues identified by the quantitative assessments, but also to further elicit information regarding possible impact on the patient's life. The interviews are semi-structured but can be flexible so that they can be guided by any issues highlighted by the quantitative data collection. The interview schedule is in Appendix A.

The interview will be conducted by the primary researcher, in a private room and last for up to one hour. The participant will be told that they can have a break or stop the interview at any time. Interviewing patients on potentially sensitive issues can lead to an emotional response, either cathartic or distressing (38). At the end of the interview there will be two questions regarding the experience of the cognitive assessments; this will provide information regarding the tolerability of performing such structured assessments in this patient group.

Analysis plan

Research question one: Are targeted therapies given within the context of Phase I trials associated with deterioration in cognitive function.

The aim of these analyses is to describe patterns of decline and explore the use of methods in this patient group. To answer the first research question and provide a description of the function of the three cognitive domains from baseline across the timepoints. The following analysis plan will be conducted:

The HVLT-R, CTMT A and B will be converted to age adjusted T scores using the professional manuals (39-42). The COWA will be converted to Z scores.

Sample means of the scores at each timepoints will be presented with standard deviations and confidence intervals. Box plots will be produced to view the data. The proportions of patients showing CI as described here will be reported as percentages. For the HVLT-R memory test CI will be defined as a T score of less than 42 (43). For the CTMT A and B CI is defined as a T score less than 40 and for the COWA CI is defined as a Z score less than -1 (44).

For peer review only - http://bmjopen.bmj.com/site/about/guidelines.xhtml

To describe the change over time and comparison of means an ANOVA repeated measures test will be conducted. Whilst caution should be taken to interpret these results due to sample size the ANOVA may help to explore possible trends in cognitive function and provide effect sizes for the changes in the dependant variables (cognitive scores). The results from the ANOVA analyses will also be used to answer research question 3, more detail will be given about the independent variables to be included in the model.

Research question 2: What is the experience of cognitive function and the impact of any deterioration on the lives of these people on Phase I trials?

To answer the quantitative aspect of this question with the objective of assessing self-reported cognitive function the following analysis will be conducted. At each timepoint the global score will be calculated according to the standard scoring protocol for FACT-cog (45). The proportions of patients reporting perceived cognitive impairment (PCI) on the PCI Subscale will be calculated at each time point using the cut-off point of >60 (46). Changes over time of PCI will be evaluated using the established MCID of 9 points difference from baseline (47).

Research Question 3: What influence do potential confounding factors of depression, anxiety and fatigue have on the cognitive scores in this patient group?

To assess the association of potential confounding variables on cognitive decline in this patient group identified variables will be included in the ANOVA repeated measures. It is known there are some relationships between anxiety and depression, sex and fatigue with CI. It is also suggested in the literature that researchers investigating cognitive function should plan to account for these possible relationships. (48). Prior to inclusion in the ANOVA correlations between the Dependant Variables and Independent Variables will be run to ensure that the model will be data driven rather than theory driven.

Bivariate correlation between baseline DV and the IV will be conducted using the Pearson R standardised correlation. This will establish the variables required to be included in the ANOVA.

Calculating effect size

Effect size analysis using Cohen d will be conducted on all statistically significant changes from baseline to help understand the practical significance of the results. A small to moderate effect size is considered meaningful.

Research Question 3 and 4

For peer review only - http://bmjopen.bmj.com/site/about/guidelines.xhtml

Proportions of recruitment, refusal and attrition rates of the study will be presented as percentages. An 70% accrual rate is selected as a level to demonstrate acceptability to patients.

Proportions of correctly completely and interpreted nurse administered cognitive assessments will be recorded as agreed by clinical psychologist reported as overall number and percentages. It is expected that 90& agreement on cognitive scores to demonstrate accuracy.

Qualitative Data Analysis

The digital audio recording will be transcribed verbatim. The data will be organised using the Nvivo software package. The premise of thematic analysis is the examination and recognition of patterns within the data that are ultimately categorised into meaningful themes (49). Thematic analysis can use different approaches and be applied to varying types of data, it may be theory driven or content driven. In this instance it will be the content in the data that will drive the analysis (50).

Integration of data

At the stage of interpretation and reporting a mixed methods matrix will be utilised as a joint display of findings. This is a suggested practical way of viewing and analysing the different types of data together and offers a tool to guide the narrative during reporting (51,52). The comprehensive results and analysis will be held in a separate database.

Patient and Public Involvement

The protocol has had patient and public involvement in the design and development stage. The protocol framework was presented and discussed at a Royal Marsden Hospital Patient and Carer Research Panel, PPI input led to decisions regarding timings and numbers of assessments. A patient representative was consulted and helped to develop the questions for the semi-structured interview.

Ethics and dissemination

The study has obtained Research Ethics Committee approval in the UK reference number: 18/LO.0084 and full Health Research Authority approval IRAS project number: 235668. The study is currently open to recruitment after a long pause in recruitment due to the COVID-19 pandemic.

Table and figures

Table 1 Research question aligned with objective, methods, and analysis
Table 2 Methods of data collection

For peer review only - http://bmjopen.bmj.com/site/about/guidelines.xhtml

Figure 1 Patient pathway aligned with Phase I trial

References

1) Wong KM, Capasso A, Eckhardt SG. The changing landscape of phase I trials in oncology. Nat Rev Clin Oncol. 2016 Feb;13(2):106-17. doi: 10.1038/nrclinonc.2015.194. Epub 2015 Nov 10. PMID: 26552953.
2) Ivanova.A. (2007) Phase I trials in oncology *Wiley Encyclopedia of clinical trials* Wiley and sons.inc. Available at https://doi.org/10.1002/9780470181218.ch1
3) National Cancer Institute (2010) Common Terminology Criteria for Adverse Events (CTCAE) https://ctep.cancer.gov/protocoldevelopment/electronic_applications/ctc.htm
4) Ng. T. Cheung. Y. Ng. Q. Ho. H and Chan. A. (2014) Vascular Endothelial Growth Factor Inhibitors and Cognitive Impairment: Evidence and Controversies. *Expert Opinion on Drug Safety* 13 (1) DOI: 10.1517/14740338.2013.82803
5) Selamat. M. Loh. S. Mackenzie. L. Vardy. J (2014) Chemobrain Experienced by Breast Cancer Survivors: A Meta-Ethnography Study Investigating Research and Care Implications *PLoS ONE* 9(9) Available at: https://doi.org/10.1371/journal.pone.0108002
6) Fitch MI, Armstrong J, Tsang S. Patients' experiences with cognitive changes after chemotherapy. Can Oncol Nurs J. 2008 Fall;18(4):180-92. English, French. doi: 10.5737/1181912x184180185. PMID: 19580104.
7) Myers.C. and Perry.J. (2012) *Cognition and Cancer* Cambridge Medicine
8) Eysenck.M and Keane.M (2010) *Cognitive Psychology: A Student's Handbook.* Sixth Edition Psychology Press
9) Harvey PD. Domains of cognition and their assessment. Dialogues Clin Neurosci. 2019 Sep;21(3):227-237. doi: 10.31887/DCNS.2019.21.3/pharvey. PMID: 31749647; PMCID: PMC6829170.
10) Ahles TA. Brain vulnerability to chemotherapy toxicities. Psychooncology. 2012 Nov;21(11):1141-8. doi: 10.1002/pon.3196. Epub 2012 Oct 1. PMID: 23023994; PMCID: PMC3788849.
11) Boykoff N, Moieni M, Subramanian SK. Confronting chemobrain: an in-depth look at survivors' reports of impact on work, social networks, and health care response. J Cancer Surviv. 2009 Dec;3(4):223-32. doi: 10.1007/s11764-009-0098-x. Epub 2009 Sep 16. PMID: 19760150; PMCID: PMC2775113.
12) Martin M, Clare L, Altgassen AM, Cameron MH, Zehnder F. Cognition-based interventions for healthy older people and people with mild cognitive impairment. Cochrane Database Syst Rev. 2011 Jan 19;(1):CD006220. doi: 10.1002/14651858.CD006220.pub2. PMID: 21249675.
13) Gehring K, Roukema JA, Sitskoorn MM. Review of recent studies on interventions for cognitive deficits in patients with cancer. Expert Rev Anticancer Ther. 2012 Feb;12(2):255-69. doi: 10.1586/era.11.202. PMID: 22316373.
14) Ganz PA. "Doctor, will the treatment you are recommending cause chemobrain?". J Clin Oncol. 2012 Jan 20;30(3):229-31. doi: 10.1200/JCO.2011.39.4288. Epub 2011 Dec 19. PMID: 22184392.
15) Deeks ED. Olaparib: first global approval. Drugs. 2015 Feb;75(2):231-40. doi: 10.1007/s40265-015-0345-6. PMID: 25616434.

For peer review only - http://bmjopen.bmj.com/site/about/guidelines.xhtml

16) Moore. H and McSherry. W. (2013) Ethical Considerations in Translational Research. *Cancer Nursing* 12(10): 22-28
17) Wefel. J. Ahles. T. Vardy. J and Schagen. S (2011) International Cognition and Cancer Task Force recommendations to harmonise studies of cognitive function in patients with cancer *The Lancet Oncology* (12)7 703-708
18) Shilling V, Jenkins V, Morris R, Deutsch G, Bloomfield D. The effects of adjuvant chemotherapy on cognition in women with breast cancer--preliminary results of an observational longitudinal study. Breast. 2005 Apr;14(2):142-50. doi: 10.1016/j.breast.2004.10.004. PMID: 15767184.
19) Schagen SB, Muller MJ, Boogerd W, Mellenbergh GJ, van Dam FS. Change in cognitive function after chemotherapy: a prospective longitudinal study in breast cancer patients. J Natl Cancer Inst. 2006 Dec 6;98(23):1742-5. doi: 10.1093/jnci/djj470. PMID: 17148777.
20) Stewart A, Collins B, Mackenzie J, Tomiak E, Verma S, Bielajew C. The cognitive effects of adjuvant chemotherapy in early stage breast cancer: a prospective study. Psychooncology. 2008 Feb;17(2):122-30. doi: 10.1002/pon.1210. PMID: 17518411.
21) Ahles TA, Saykin AJ, McDonald BC, Li Y, Furstenberg CT, Hanscom BS, Mulrooney TJ, Schwartz GN, Kaufman PA. Longitudinal assessment of cognitive changes associated with adjuvant treatment for breast cancer: impact of age and cognitive reserve. J Clin Oncol. 2010 Oct 10;28(29):4434-40. doi: 10.1200/JCO.2009.27.0827. Epub 2010 Sep 13. PMID: 20837957; PMCID: PMC2988635.
22) Collins B, MacKenzie J, Tasca GA, Scherling C, Smith A. Cognitive effects of chemotherapy in breast cancer patients: a dose-response study. Psychooncology. 2013 Jul;22(7):1517-27. doi: 10.1002/pon.3163. Epub 2012 Aug 30. PMID: 22936651.
23) Collins B, Mackenzie J, Tasca GA, Scherling C, Smith A. Persistent cognitive changes in breast cancer patients 1 year following completion of chemotherapy. J Int Neuropsychol Soc. 2014 Apr;20(4):370-9. doi: 10.1017/S1355617713001215. Epub 2013 Nov 15. PMID: 24229809.
24) Jones D, Vichaya EG, Wang XS, Sailors MH, Cleeland CS, Wefel JS. Acute cognitive impairment in patients with multiple myeloma undergoing autologous hematopoietic stem cell transplant. Cancer. 2013 Dec 1;119(23):4188-95. doi: 10.1002/cncr.28323. Epub 2013 Sep 16. PMID: 24105672; PMCID: PMC3834212.
25) Cruzado J. Lopez- Santiago S. Martinez-Marin V. Jose-Moreno G. Custadio AB and Feliu J (2014) Longitudinal Study of cognitive dysfunctions induced by adjuvant chemotherapy in colon cancer patients. *Supportive Cancer Care* 22(7): 1815-1823
26) Kohli S, Griggs JJ, Roscoe JA, Jean-Pierre P, Bole C, Mustian KM, Hill R, Smith K, Gross H, Morrow GR. Self-reported cognitive impairment in patients with cancer. J Oncol Pract. 2007 Mar;3(2):54-9. doi: 10.1200/JOP.0722001. PMID: 20859374; PMCID: PMC2793748.
27) Quesnel C, Savard J, Ivers H. Cognitive impairments associated with breast cancer treatments: results from a longitudinal study. Breast Cancer Res Treat. 2009 Jul;116(1):113-23. doi: 10.1007/s10549-008-0114-2. Epub 2008 Jul 16. Erratum in: Breast Cancer Res Treat. 2009 Jul;116(1):129-30. PMID: 18629633.
28) Klaver KM, Duijts SFA, Engelhardt EG, Geusgens CAV, Aarts MJB, Ponds RWHM, van der Beek AJ, Schagen SB. Cancer-related cognitive problems at work: experiences of survivors and professionals. J Cancer Surviv. 2020 Apr;14(2):168-178. doi: 10.1007/s11764-019-00830-5. Epub 2019 Nov 25. PMID: 31768861; PMCID: PMC7182611.
29) Tricco AC, Lillie E, Zarin W, O'Brien KK, Colquhoun H, Levac D, Moher D, Peters MDJ, Horsley T, Weeks L, Hempel S, Akl EA, Chang C, McGowan J, Stewart L, Hartling L, Aldcroft A, Wilson MG, Garritty C, Lewin S, Godfrey CM, Macdonald MT, Langlois EV, Soares-Weiser K, Moriarty J, Clifford T, Tunçalp Ö, Straus SE. PRISMA Extension for Scoping Reviews (PRISMA-ScR): Checklist and Explanation. Ann Intern Med. 2018 Oct 2;169(7):467-473. doi: 10.7326/M18-0850. Epub 2018 Sep 4. PMID: 30178033.

For peer review only

30) Hlubocky FJ, Sachs GA, Larson ER, Nimeiri HS, Cella D, Wroblewski KE, Ratain MJ, Peppercorn JM, Daugherty CK. Do Patients with Advanced Cancer Have the Ability to Make Informed Decisions for Participation in Phase I Clinical Trials? J Clin Oncol. 2018 Aug 20;36(24):2483-2491. doi: 10.1200/JCO.2017.73.3592. Epub 2018 Jul 9. PMID: 29985748; PMCID: PMC6097830.
31) Jones RL, Serrano C, von Mehren M, George S, Heinrich MC, Kang YK, Schöffski P, Cassier PA, Mir O, Chawla SP, Eskens FALM, Rutkowski P, Tap WD, Zhou T, Roche M, Bauer S. Avapritinib in unresectable or metastatic PDGFRA D842V-mutant gastrointestinal stromal tumours: Long-term efficacy and safety data from the NAVIGATOR phase I trial. Eur J Cancer. 2021 Mar;145:132-142. doi: 10.1016/j.ejca.2020.12.008. Epub 2021 Jan 16. PMID: 33465704.
32) McKay RR, De Velasco G, Werner L, Bellmunt J, Harshman L, Sweeney C, Rosenberg JE, Hirsch M, Signoretti S, Van Allen EM, Walsh M, Vaishampayan U, McDermott DF, Choueiri TK. A phase 1 study of buparlisib and bevacizumab in patients with metastatic renal cell carcinoma progressing on vascular endothelial growth factor-targeted therapies. Cancer. 2016 Aug 1;122(15):2389-98. doi: 10.1002/cncr.30056. Epub 2016 May 19. PMID: 27198170; PMCID: PMC5567751.
33) Wicki A, Brown N, Xyrafas A, Bize V, Hawle H, Berardi S, Cmiljanović N, Cmiljanović V, Stumm M, Dimitrijević S, Herrmann R, Prêtre V, Ritschard R, Tzankov A, Hess V, Childs A, Hierro C, Rodon J, Hess D, Joerger M, von Moos R, Sessa C, Kristeleit R. First-in human, phase 1, dose-escalation pharmacokinetic and pharmacodynamic study of the oral dual PI3K and mTORC1/2 inhibitor PQR309 in patients with advanced solid tumors (SAKK 67/13). Eur J Cancer. 2018 Jun;96:6-16. doi: 10.1016/j.ejca.2018.03.012. Epub 2018 Apr 13. PMID: 29660598.
34) Basch E, Jia X, Heller G, Barz A, Sit L, Fruscione M, Appawu M, Iasonos A, Atkinson T, Goldfarb S, Culkin A, Kris MG, Schrag D. Adverse symptom event reporting by patients vs clinicians: relationships with clinical outcomes. J Natl Cancer Inst. 2009 Dec 2;101(23):1624-32. doi: 10.1093/jnci/djp386. Epub 2009 Nov 17. PMID: 19920223; PMCID: PMC2786917.
35) Veitch ZW, Shepshelovich D, Gallagher C, Wang L, Abdul Razak AR, Spreafico A, Bedard PL, Siu LL, Minasian L, Hansen AR. Underreporting of Symptomatic Adverse Events in Phase I Clinical Trials. J Natl Cancer Inst. 2021 Aug 2;113(8):980-988. doi: 10.1093/jnci/djab015. PMID: 33616650; PMCID: PMC8502480.
36) Sailors MH, Bodurka DC, Gning I, Ramondetta LM, Williams LA, Mendoza TR, Agarwal S, Sun CC, Cleeland CS. Validating the M. D. Anderson Symptom Inventory (MDASI) for use in patients with ovarian cancer. Gynecol Oncol. 2013 Aug;130(2):323-8. doi: 10.1016/j.ygyno.2013.05.009. Epub 2013 May 15. PMID: 23685012; PMCID: PMC3713195.
37) Upadhayay N, Guragain S. Comparison of cognitive functions between male and female medical students: a pilot study. J Clin Diagn Res. 2014 Jun;8(6):BC12-5. doi: 10.7860/JCDR/2014/7490.4449. Epub 2014 Jun 20. PMID: 25120970; PMCID: PMC4129348.
38) Ryan, Francis; Coughlan, Michael & Cronin, Patricia (2009). *Interviewing in qualitative research: The one-to-one interview.* International Journal of Therapy and Rehabilitation, *16*(6), 309
39) Brandt. J. and Benedict.R. (2001) Hopkins Verbal Learning Test-Revised- Professional Manual Psychological Assessment Resources
40) Benton A, Hamsher Brandt. J. and Benedict.R. (2001) Hopkins Verbal Learning Test-Revised- Professional Manual Psychological Assessment Resources
41) Benton, Hamsher and Sivan. A (1978) Multilingual Aphasia Examination Manual of Instructions 3rd Edition Psychological Assessment Resources
42) Reynolds C (2002) Comprehensive Trail-Making Test Examiners Manual Pro-Ed
43) Hirotake. S et al (2016) Factors Affecting the Baseline and Post-Treatment Scores on the Hopkins Verbal Learning Test-Revised Japanese Version before and after whole brain

For peer review only - http://bmjopen.bmj.com/site/about/guidelines.xhtml

radiation therapy International journal of Molecular Sciences, 17(11) p.1834 https://search.ebscohost.com/login.aspx?direct=true&db=edb&AN=119771439&site=eds-live(Accessed:7November 2021)

44) Vardy JL, Stouten-Kemperman MM, Pond G et al. (2019) A mechanistic cohort study evaluating cognitive impairment in women Imaging Behav 13(1): 15treated for breast cancer. Brain–26.
45) Cella DF, Tulsky DS, Gray G, Sarafian B, Linn E, Bonomi A, Silberman M, Yellen SB, Winicour P, Brannon J, et al. The Functional Assessment of Cancer Therapy scale: development and validation of the general measure. J Clin Oncol. 1993 Mar;11(3):570-9. doi: 10.1200/JCO.1993.11.3.570. PMID: 8445433.
46) Dyk KV, Crespi CM, Petersen L, Ganz PA. Identifying Cancer-Related Cognitive Impairment Using the FACT-Cog Perceived Cognitive Impairment. JNCI Cancer Spectr. 2019 Nov 29;4(1):pkz099. doi: 10.1093/jncics/pkz099. PMID: 32064458; PMCID: PMC7015054.
47) Cheung YT, Foo YL, Shwe M, Tan YP, Fan G, Yong WS, Madhukumar P, Ooi WS, Chay WY, Dent RA, Ang SF, Lo SK, Yap YS, Ng R, Chan A. (2014) *Minimal clinically important difference (MCID) for the functional assessment of cancer therapy: cognitive function (FACT-Cog) in breast cancer patients.* J Clinical Epidemiology 67(7):811-20. doi: 10.1016/j.jclinepi.2013.12.011. Epub 2014 Mar 18. PMID: 24656406.
48) Lange, F. Joly, J Vardy, G. Winocur, M.B. De Ruiter, H. Castel *(2019) Cancer-related cognitive impairment: an update on state of the art, detection, and management strategies in cancer survivors* Annals of Oncology *(30) 12 P1925-1940 DOI: https://doi.org/10.1093/annonc/mdz410*
49) Guest. G. (2012) *Applied Thematic Analysis* Thousand Oaks California
50) Braun V and Clarke V (2006) Using thematic analysis in psychology. *Qualitative Research in Psychology* 3(2): 77-101
51) Johnson R. Onwuegbuzie A and Turner. L. (2007) Toward a definition of mixed methods research. *Journal of mixed methods* 1(2): 112-133
52) Onwuegbuzie AJ, Slate JR, Leech NL, Collin. N Kathleen. M (2009) *Mixed data analysis: Advanced integration techniques.* International Journal of Multiple Research Approaches 3(1): 13–33.

Author's Contributions

Sarah Stapleton – Primary Researcher, Research design concept, Protocol Development and Writing

Prof. Anne-Sophie Darlington- PhD primary supervisor, Protocol Development

Prof. Johann De-Bono – Clinical oversight of the study as head of unit, review of protocol development

Prof. Theresa Wiseman – Principal Investigator , PhD supervisor, Protocol development ,

Competing Interests

Sarah Stapleton None declared however SS post is funded by Cancer Research UK

Prof. Anne-Sophie Darlington None declared

For peer review only - http://bmjopen.bmj.com/site/about/guidelines.xhtml

Prof. Theresa Wiseman None declared

Prof. Johann De-Bono: JDB has served on advisory boards and received fees from many companies including Astra Zeneca, Astellas, Bayer, Bioxcel Therapeutics, Boehringer Ingelheim, Cellcentric, Daiichi, Eisai, Genentech/Roche, Genmab, GSK, Janssen, Merck Serono, Merck Sharp & Dohme, Menarini/Silicon Biosystems, Orion, Pfizer, Qiagen, Sanofi Aventis, Sierra Oncology, Taiho, Vertex Pharmaceuticals. He is an employee of The ICR, which have received funding or other support for his research work from AZ, Astellas, Bayer, Cellcentric, Daiichi, Genentech, Genmab, GSK, Janssen, Merck Serono, MSD, Menarini/Silicon Biosystems, Orion, Sanofi Aventis, Sierra Oncology, Taiho, , Pfizer, Vertex, and which has a commercial interest in abiraterone, PARP inhibition in DNA repair defective cancers and PI3K/AKT pathway inhibitors (no personal income). He was named as an inventor, with no financial interest, for patent 8,822,438. JDB is a National Institute for Health Research (NIHR) Senior Investigator. The views expressed in this article are those of the author(s) and not necessarily those of the NHS, the NIHR, or the Department of Health.

Funding Statement

The research received no specific grant from any funding agency in the public, commercial or not for profit sectors. However the primary researcher’s post is funded by Cancer Research UK.

Acknowledgements

The author would like to acknowledge the Royal Marsden Patient and Carer Research Review Panel for their contribution to the protocol development.

-

For peer review only

For peer review only - http://bmjopen.bmj.com/site/about/guidelines.xhtml

Figure 1 Patient pathway on study aligned with Phase I trial

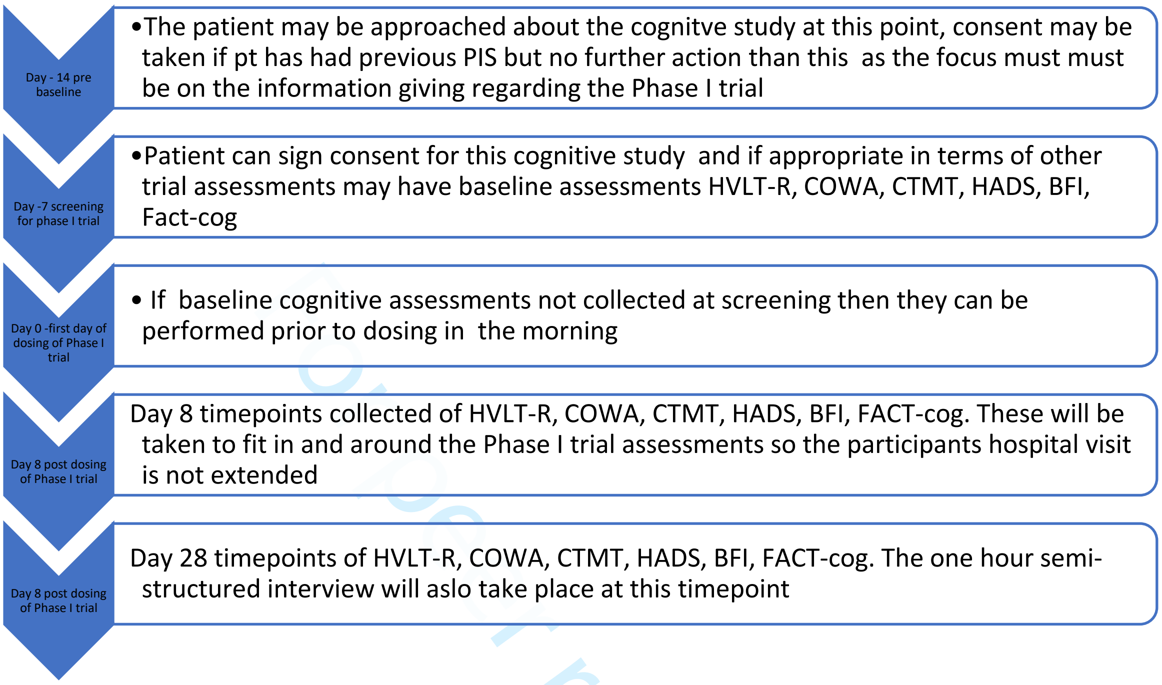

For peer review only

Appendix A Interview Schedule

Interviewer to introduce themselves to the participant, clarify that they know why they are here and that consent still stands. Give examples of phrasing of questions and suggested idea of how long the interview will last. Remind the participant that they can stop the process if they wish. Recheck that they are happy for the audio recording to start, and they are ready to begin the interview.

Using open ended questions, the Interview will cover the following at least but not exclusively the following areas:

Memory

Concentration

Ability to speak/articulate/communicate

General Health

Impact of trial treatment/schedule

Mood

The first two questions will be general to help the patient to start talking and feel relaxed in the environment

1) Tell me how you have been since you started the trial

2) Can you tell me of any changes you have noticed since starting the trial?

If the participant mentions any cognitive or related symptoms, they will be asked to expand on this, using such phrases such as

- **"**Can you tell me a bit more about that?"
- "What was that like for you?"
- "How did you deal with that? "

If not questions regarding domains of cognitive function will be asked – examples of this include:

"Tell me about how your memory has been since you started the trial"

"tell me how your concentration has been since starting on the trial? "

For peer review only

"Have you noticed any differences in speaking or finding the right words sometimes?"

"Have you noticed any changes in your ability to think straight or work things out?"

"Have you had any difficulty relating to this? "

"How has your mood been since starting on the trial drug?"

If any changes or symptoms are discussed ask patient to elaborate – when did it start, how does it manifest/feel/ present? – Characterise the symptom with the participant onset, severity, duration, improvements, deterioration etc. Examples of questions to elaborate on symptoms are:

"How did you first notice this?"

"Tell me a bit about what happened to you "

"How did you feel when this was happening?"

Other questions will then cover the areas that are relevant to the investigation of this study. If patients have discussed specific symptoms the following questions will be asked in relation to this.

"Tell me how this has impacted on your life?"

"Has this affected you at work/home/ your relationships with others in any way?"

Once the impact and effect on certain areas has been established try to prompt further to explore how this made the patient feel and how they cope with this.

"Did you try anything to help the situation? "

"Was this a difficult thing to cope with?"

Dependant on what the interviewee discusses during the interview, complete with an exploration of strategies or useful interventions that have or may have helped covering:

"Would they have been anything useful to know about pre- treatment to help you prepare for any of these symptoms?"

"Tell me about any strategies that you have used yourself to help you overcome these problems?"

"tell me of anything the team here could have done to help you manage the ……. You discussed before "

For peer review only

The last two questions are regarding the acceptability of the assessments performed for this study- these questions will be asked to all participants.

"How have you found the extra cognitive assessments that have been performed for this study?"

"Would you be happy to have these assessments performed alongside treatment as the norm?"

End the interview by checking if there is anything that the participant wants to add. Ask if they are happy for the interview to be used. Thank the participant for taking part.

For peer review only
